# Toward autonomous wearable triboelectric systems integrated on textiles

**DOI:** 10.1016/j.isci.2022.105264

**Published:** 2022-10-04

**Authors:** Valentin Gaubert, Gaëtan Vauche, Jennifer Weimmerskirch-Aubatin, Christophe Corbier, Xavier Boddaert, Roger Delattre, Thierry Djenizian

**Affiliations:** 1 Mines Saint-Etienne, Center of Microelectronics in Provence, Department of Flexible Electronics, 13541 Gardanne, France; 2 Saint-Etienne Jean Monnet University, Roanne Technology University Institute, University of Lyon, LASPI (EA3059), 42334 Roanne, France; 3 SOFILA, 26 rue Henri GORJUS 69004 Lyon, France; 4 Al-Farabi Kazakh National University, Center of Physical-Chemical Methods of Research and Analysis, Tole bi str., 96A., Almaty, Kazakhstan

**Keywords:** Biodevices, Bioelectronics, Energy Resources, Electronic materials

## Abstract

One of the major requirements of smart textiles is to achieve the integration of an energy source for powering embedded electronic systems. In this context, textile triboelectric nanogenerators (T-TENGs) are particularly well suited to imperceptibly play this role in the core of textiles, making them highly appealing for the development of future autonomous systems. This article reviews the wide range of topics related to T-TENGs technology starting from triboelectric generation (textile device and behavior modeling) up to the complete integration of power transfer (rectifier) circuits on textiles. The modeling part deals with the current mathematical models of the triboelectric charge transfer in order to highlight efficient power transfer circuits. Then the materials and architectures used to fabricate different types of T-TENGs are described. Finally, the methods and technologies to seamlessly integrate the power transfer circuit into textiles are discussed: from realizing electrically conductive tracks through to integrating electronic component on textiles.

## Graphical abstract

**Figure:**
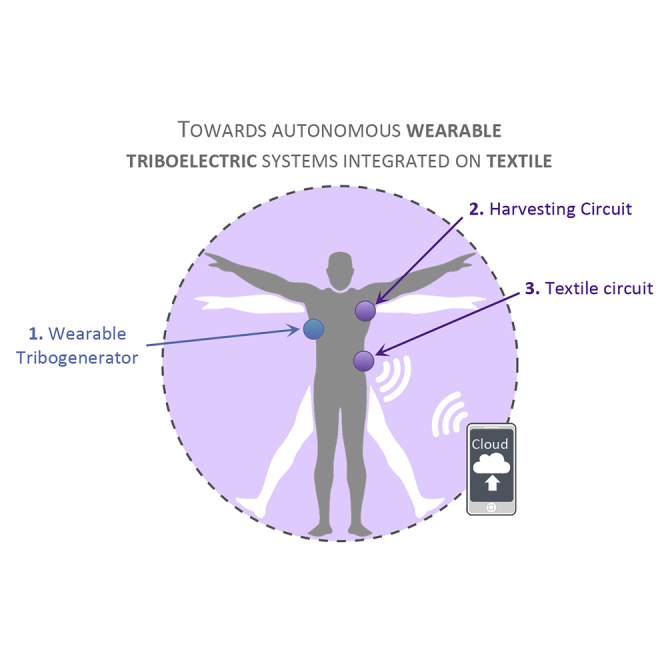

## Introduction

In the field of wearable technologies, the smart textiles domain has been in constant evolution as its appearance at the end of the 1980s, as new functionalities were added. At first, the term only concerned so-called passive smart textiles, with shape-memory polymers capable of reacting to stimuli. As time went on, the integration of electronic components enabled the development of increasingly intelligent textiles, integrating more and more complex functions, particularly integrating physiological monitoring sensors. The opportunity to use this type of device in a large number of applications and markets, such as sportswear, medical monitoring or integration into certain composites, has led to market forecasts in the 2010s predicting a rapid take off of this market in the next 5-10 years. However, forecasts are constantly being revised downwards and promising projects are struggling to move beyond the prototype or small series stages (Schwarz et al., 2010) (Sugathan and Hendry, 2017). There are two main points explaining the lack of success of this type of system with the general public. Firstly, a problem of limited autonomy, which requires regular recharging or replacing the batteries of these systems. Secondly, an imperfect integration of the various functionalities, such as the presence of a rigid part for the power supply and sometimes fragile interconnections, requiring a particular attention from the user in order to not damage the device. These two previous points cause a lack of comfort when wearing and during the user experience that will discourage the latter from investing in a more expensive and restricitive device although offering new possibilities.

All these points make users tend to prefer wearables such as smartwatches to have more features, such as physiological monitoring and wireless communication systems. However, the small area covered by these systems limits the variety of physiological signals of interest that can be measured. It is therefore necessary to develop electronic systems integrated into the textile, eliminating the need to recharge the energy storage system using wires, and for which we retain the comfort and breathability of a traditional textile.

The advent of nanogenerators and more specifically triboelectric nanogenerators (TENGs) as early as 2012 (Fan et al., 2012) has opened prospects for harvesting power from human motions and directly embedded into the textile, eliminating the need for external charging.

This review aims to bring global understanding of wearable textile TENGs (T-TENGs) to specialists of three different fields: (i) comprehensive study of triboelectricity phenomenon, (ii) textile integration of needed materials, and (iii) electronics integration onto textiles of power transfer circuits for embedded applications. The operation of TENG and physical origins of the phenomenon are presented in the first section. Section generalities describes main theoretical approaches such as Gauss theorem and Maxwell’s theory. Different materials and structures tested as T-TENGs are shown in Section theoretical approach of TENGs. Section t-TENGs covers the different way of making textile conductive tracks before discussing different methods to integrate electronic components or circuits to textiles. Conclusions and perspectives are drawn in Section electronics system integration on textiles.

## Generalities

This section will address main phenomena of energy harvesting devices and difficulties to distinguish their electrical signal. Among these phenomena, triboelectricity and TENG will be developed.

### Main phenomena to energy harvesting

Energy harvesting devices arouse a wide interest for the conversion of mechanical to electrical energy and applications cover a wide range of domains from micrometer-scale vibration to human motions. There exists many phenomena to generate electric charges. Piezoelectricity, triboelectricity and ferroelectricity remain widely used in systems. Piezoelectric effect is the ability of certain materials to generate electric charges on the surface under pressure/strain. Triboelectricity is the phenomenon of contact electrification between two materials becoming electrically charged upon contact or friction. Ferroelectricity is a characteristic of certain materials that have a spontaneous electric polarization that can be reversed by the application of an external electric field (Wu and Li, 2021). However, the electrical signals generated by piezoelectricity, triboelectricity and ferroelectricity are difficult to distinguish. Indeed under practical testing or application conditions, the piezoelectric and triboelectric processes occur nearly simultaneously and their contributions are indistinguishable.

As explained in (Suo et al., 2016), the authors showed that the difference was reflected only by the overall intensity of the electric signal peaks. To identify the piezoelectric and triboelectric contributions in a mechanical energy conversion process, the authors designed a cantilever-type resonator using a composite film that was partially covered by electrodes. More recently, a deep work related to piezoelectric polymers is emerging as exceptionally promising materials for energy harvesting (Šutka et al., 2020). The difference in mechanism of energy generation/harvesting has implications for how piezoelectric generator (PEG) or triboelectric generator (TEG) systems are deployed. The output of each of them is measured in voltage, current, power and charge. The fundamental process generating the output is extremely important. Many researchers assume that they are only measuring piezoelectric output. In fact, the triboelectric output is often misreported as a piezoelectric output, resulting in extremely high piezoelectric coefficients. The authors concluded on the need to address overlap of piezoelectric with triboelectric effects and provide a measurement protocol and guidance.

### Triboelectric nanogenerators

A relevant investigation field of triboelectric effect is related to TENGs. In (Lu et al., 2019), the authors proposed a new method which exploited energy oscillation between the TENG and an inductive load to boost the energy output for the TENG with contact-separation (CS) mode. A review has been presented in (Chen et al., 2020c) based on polymer materials (PM) for high performance TENGs. Today, there are efforts to improve the performance of TENGs through methods such as tribosurface morphology engineering, surface molecular functionalization and bulk composition modification. The performance of TENGs depends on generated triboelectric surface charges $\sigma_{T}$ during the friction process.

**Figure 1:**
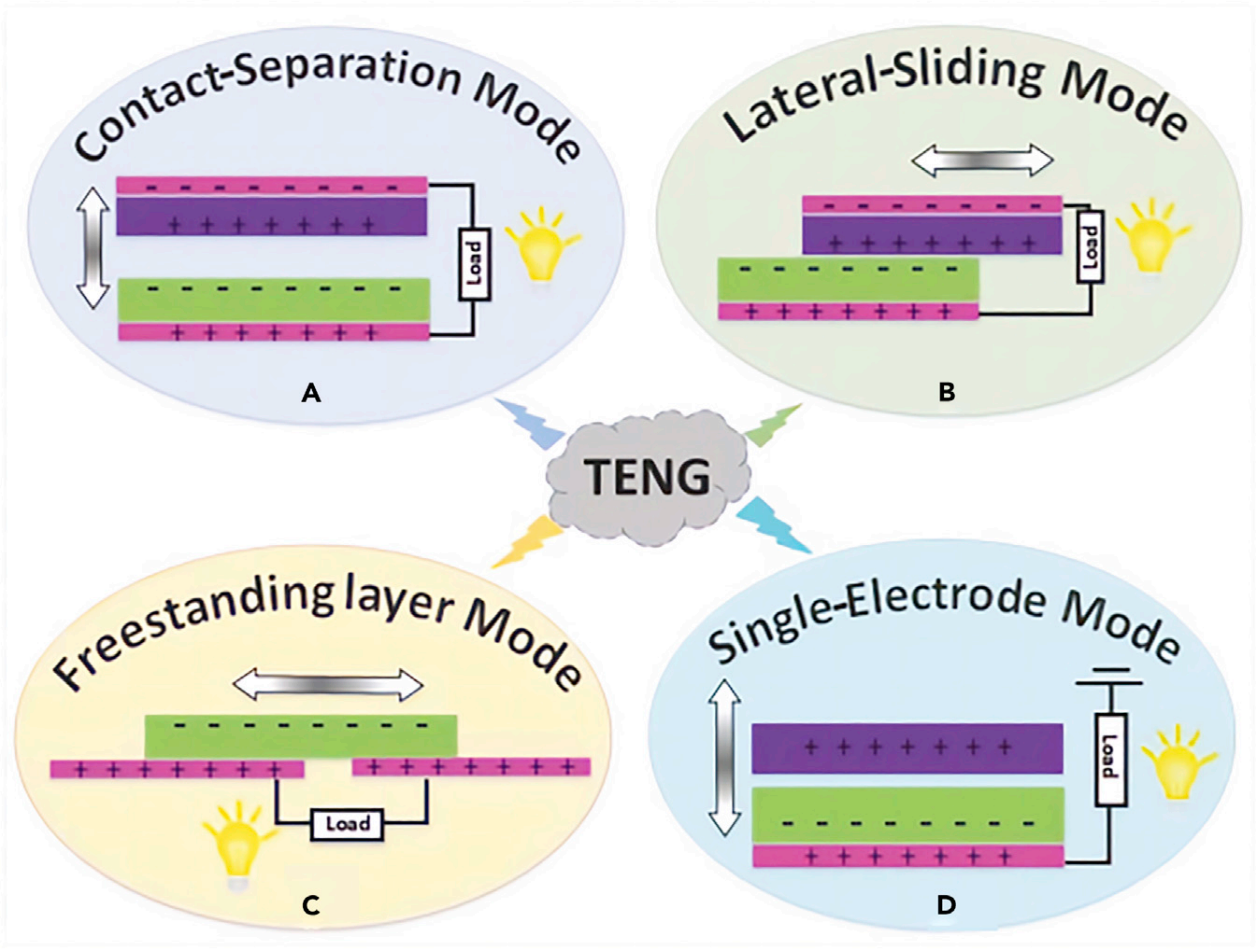
Scheme of the 4 different working modes of triboelectric nanogenerators Reproduced with permission from (Ahmed et al., 2019). Creative Commons CC-BY license.

There are four different functionings depending on the design of TENGs. In the case of CS mode (Figure 1A), there is a vertical intermittent contact between two dielectric surfaces, each of them being linked to an electrode of the system. For lateral-sliding mode (Figure 1B), the displacement is horizontal and there is a rubbing between the two dielectric surfaces. As for the freestanding layer mode (Figure 1C), both electrodes have the same dielectric, and the other dielectric goes between electrodes to create an alternative current (AC). Finally, for the single electrode mode (Figure 1D), as it is noted by its name, there is only one electrode linked to one of the dielectrics, the other end of the electric circuit corresponding to the ground.

As for the physical origin of the phenomenon, it is a combination of contact electrification and electric induction. The contact electrification corresponds to the fact that when two dielectric layers are put into contact and then separated, both will have a nonneutral surface charge: one will be positively charged, while the other will be negatively charged.

**Figure 2:**
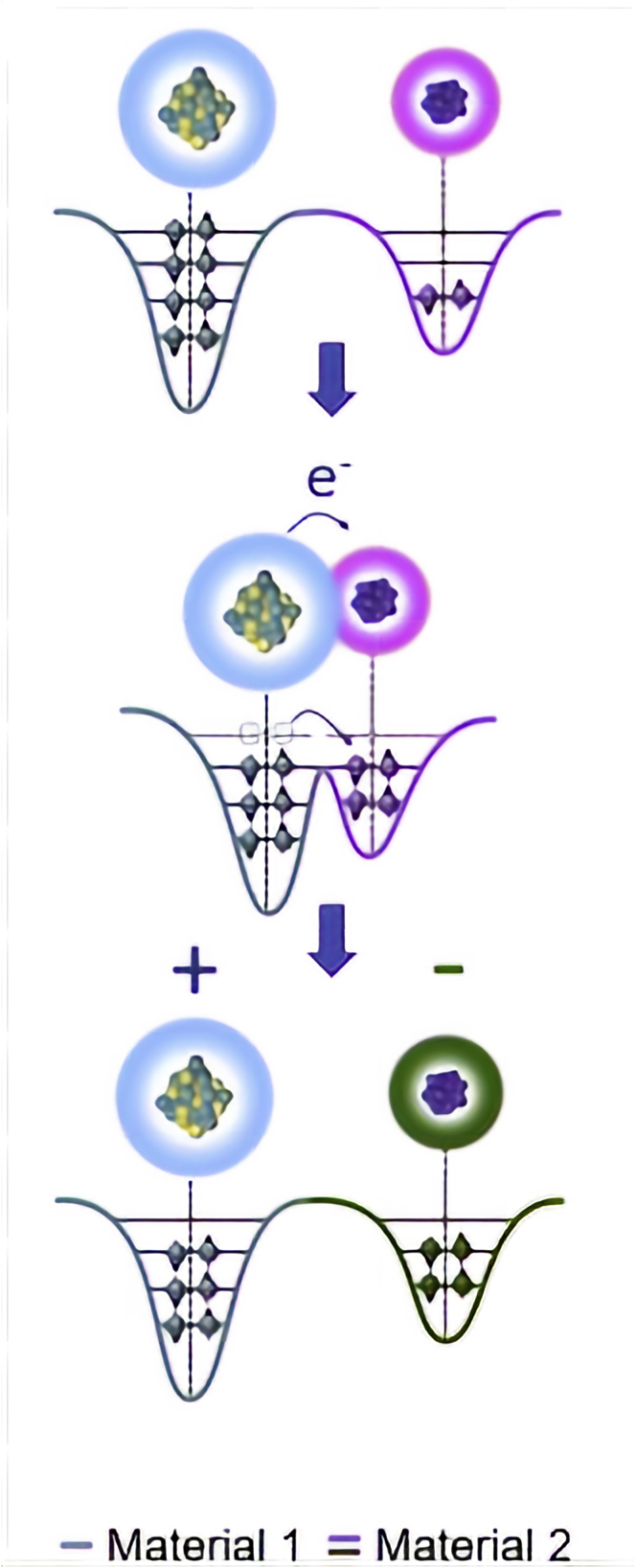
The atomic-scale-electron-cloud-potential-well model to describe the contact electrification process of a TENG Reproduced with permission from (Zhou et al., 2020). Creative Commons CC-BY license.

One of the first explanations of contact electrification for metal-metal and metal-semiconductor has been established in the framework of the band theory (Vick, 1953). This article highlights the significance of the surface states upon surface electrification and also mentions that the charge transfer can occur by ionic transport in few cases. With the rise of TENGs that promise electricity generation through these phenomena, the detailed study of these phenomena with a more detailed quantum approach has developed in recent years, proposing an atomic scale charge transfer mechanism (Wang and Wang, 2019). As depicted in Figure 2, when there is a contact between the two materials, the electronic clouds can overlap to form a bond. In this case, the two separate potential wells result in one double-well potential, with a lower energy barrier between the two wells. An electronic transfer can then occur from one well to the other. Once the materials are separated, the transferred electrons stay trapped as static charges on the surface (negatively charged), while the other surface stays depleted of electrons (positively charged). Another comprehension of the system can be gained by studying the surface electrons as an open quantum system linked to the bulk systems (Alicki and Jenkins, 2020).

In order to have a better overview of the dynamics of contact electrification, there is a regained interest in experiments dedicated to the comprehension of fine mechanisms of contact electrification. Measurements of contact electrification of nonmetallic inorganic materials (Zou et al., 2020) confirm the origin of triboelectricity as coming from a quantum electronic transfer. Electrons go to the lowest energy available electronic state once the two electron clouds overlap.

The study of temperature dependency of this phenomenon (Wang et al., 2020) also confirms the mainly electronic origin, at least in the case of solid-solid contact. The study of the dynamics of contact electrification in the case of two metallic spheres (Kaponig et al., 2021) reveals that electron transfer happens just during the contact in a 6-8 μs time span, confirming the need of a contact for the electrification to happen.

The growing body of evidence is converging toward the fact that electronic transfer is the main driver of surface charge transfer occurring during contact electrification.

The other phenomenon playing a role in the mechanism of TENG is the electric induction. When the two dielectrics are in contact, there is a global neutral charge and the system is at equilibrium. Once the two parts of the system are moved away from each other, there is an electric charge at the surface of the dielectric, inducing a charge flow between electrodes in exterior circuit in order to preserve a global charge neutrality. Once the two electrodes are far enough one from the other, there is no current in the circuit, as the electrostatic equilibrium is restored. When the two electrodes are brought back closer, a current in the opposite direction of the previous one emerges, until the dielectrics are back to contact, as the original situation. A continuous movement will then generate an alternative current. In the next section, a more detailed approach of the electric modeling is given.

## Theoretical approach of triboelectric nanogenerators

This section will develop two approaches of TENG modeling from Gauss theorem and Maxwell electromagnetic theory. Then optimal impedance will be given for these approaches. Eventually, energy transfer device and its impedance matching will be discussed.

### Modeling

Triboelectric investigations require a relevant knowledge of physical phenomena and theory (Lu et al., 2019; Chen et al., 2020c). Among those investigations the research of electrostatic induction is presented in (Chen et al., 2020a) and the authors suggested a novel universal numerical method capable of calculating dynamic behavior of TENGs with complex 2D/3D geometries under practical movements using finite element method (FEM) based moving mesh method and air buffer layers.

Investigations and applications are resulting from theoretical studies of contact-mode TENGs as an effective power source. Based on the charge separation mechanism, TENGs can be divided into two categories: contact-mode based on vertical charge polarization and lateral-mode (or sliding-mode) based on in-plane charge polarization. For more detail, the reader could refer to (Henniker, 1962; Lacks, 2012; Fan et al., 2012; Shen et al., 2016; Pan and Zhang, 2017; Choi et al., 2017; Xu et al., 2018; Wang and Wang, 2019; Lacks and Shinbrot, 2019; Feshanjerdi and Malekan, 2019; Zou et al., 2019; Lin et al., 2019; Kulbago and Chen, 2020).

**Figure 3:**
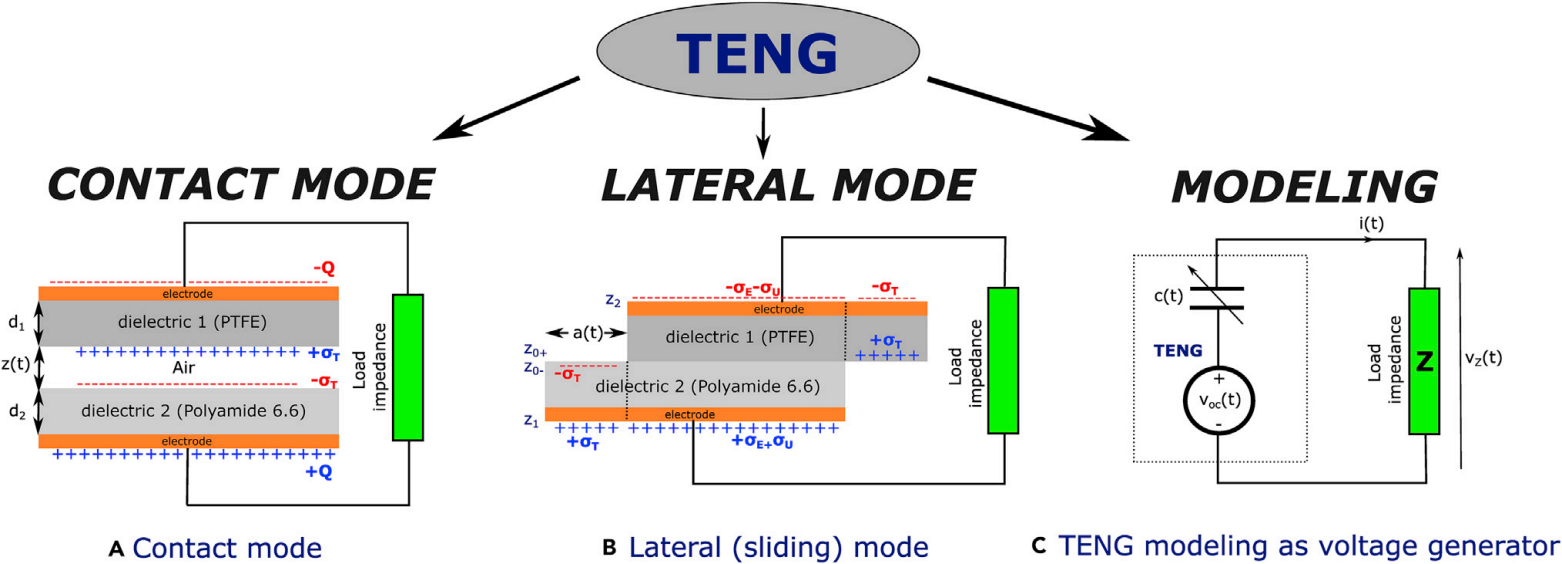
Schematic of different working modes and modeling of TENG (A) Theoretical models for dielectric-to-dielectric contact-mode TENG, (B) Theoretical models for dielectric-to-dielectric sliding-mode TENG, (C) Electric model of TENG device connected to load impedance.

A first approach has been studied from Gauss theorem (Niu et al., 2013) in order to obtain the electric field *E* and transferred surface charges *Q* to deduce current and voltage in load resistance. In this work, theoretical models such as dielectric-to-dielectric contact-mode TENG and conductor-to-dielectric contact-mode TENG were introduced (Figure 3A). The authors showed that the voltage between two electrodes is

(Equation 1) $$V\left(t\right)=\frac{-Q}{S.\varepsilon_{0}}\left(d_{0}+z\left(t\right)\right)+\frac{\sigma_{T}.z\left(t\right)}{\varepsilon_{0}}$$

where *z*(*t*) is the relative displacements between two dielectrics, *σ*_*T*_ the triboelectric surface charge density, and *S* the surface of dielectric. The open circuit voltage proportional to *σ*_*T*_ and *z*(*t*) is

(Equation 2) $$V_{oc}\left(t\right)=\;\frac{\sigma_{T}.z\left(t\right)}{\varepsilon_{0}}$$

This implies an optimal contact electrification between dielectrics and electrodes to attain a sufficient voltage for practical uses. The Gauss approach seems to give a good approximation of voltage and charge but remains approximate.

A more complex study based on electromagnetic field has been developed. This one has been reported in (Shao et al., 2019; Shao et al., 2020a; Shao et al., 2020b; Shao et al., 2020c) from Maxwell’s approach using displacement current as the driving force to convert mechanical energy of motions into electricity for vertical and lateral contact-modes. Maxwell modeling improved the Gauss approach in-depth. Based on a set of finite-sized charged planes (FSCP), a simple time-dependent three-dimensional spatial model for the electric potential and electric field in an inhomogeneous medium composed of dielectric materials and metal contacts is proposed and used to assert TENG operation. This approach is based on electric displacement **D** given by

(Equation 3) $$D=\varepsilon_{0}\varepsilon_{r}E$$

Where $\varepsilon_{0}$ is the vacuum permittivity and $\varepsilon_{r}$ the relative permittivity of dielectric material. The corresponding displacement current density is $J_{D}=\frac{dD}{dt}$ and the displacement current is given by $i_{D}=\int J_{D}\cdot dS$, which only accounts for the polarization in the medium owing to the presence of an electric field. However, considering the presence of electrostatic charges on surfaces owing to the piezoelectric or triboelectric effect, Wang in (Shao et al., 2019) proposed to add a new term P in Equation 3 related to the nonelectric field-induced polarization owing to mechanical triggering (contact electrification). The integral solution of the Maxwell-Poisson equation in Cartesian coordinates r=(x,y,z) given by ∇·D(r)=ρ(r) leads to the electric potential φ(r) given by

(Equation 4) $$\varphi \left(r\right)=\frac{1}{4\pi \varepsilon \left(r\right)}\int \frac{\rho \left(r^{\prime }\right)dV}{\left|r-r^{\prime }\right|}$$

where ε(r) is the permittivity in the medium and ρ the charge density. The N-finite-sized planes approach leads to a quasielectrostatic 3D model (Shao et al., 2019) when part of the charged planes move with a low frequency and a change in total electric field occurs. On the other hand, low frequency and linear motions are considered in order to simplify calculations, meaning that magnetic effects are null. Electric field E and electric potential φ depend on cartesian coordinates r=(x,y,z) leading to

(Equation 5) $$E\left(r,t\right)=-\frac{\partial \varphi \left(r,t\right)}{\partial x}e_{\text{x}}-\frac{\partial \varphi \left(r,t\right)}{\partial y}e_{\text{y}}-\frac{\partial \varphi \left(r,t\right)}{\partial z}e_{\text{z}}$$

where $e_{i}$ are unit vectors in the orthonormal basis.

In vertical modeling, some symmetries in x and y leads to simply above expression of electric field as $E^{Z}\left(r,t\right)=-\frac{\partial \varphi \left(r,t\right)}{\partial z}e_{z}=\sum_{i\in E}E_{i}^{Z}\left(r,t\right)$ with

(Equation 6) $$E_{i}^{Z}\left(r,t\right)=\frac{\sigma_{i}\left(t\right)}{4.\pi .\varepsilon \left(r\right)}\underset{S}{\iint }\frac{\left(z-z_{i}\left(t\right)\right)dx^{\text{′}}dy^{\text{′}}}{{\left[{\left(x-x^{\text{′}}\right)}^{2}+{\left(y-y^{\text{′}}\right)}^{2}+{\left(z-z_{i}\left(t\right)\right)}^{2}\right]}^{3/2}}$$

The surface charge density is $\sigma_{i}\left(t\right)$ at the vertical position $z_{i}\left(t\right)$ and S=ab the dielectric surfaces. When electrodes are connected to load impedance Z, transferred surface charge density $\sigma_{U}\left(t\right)$ is generated and the differential equation in $\sigma_{U}\left(t\right)$ is

(Equation 7) $$-ZS\frac{d\sigma_{U}\left(t\right)}{dt}=\varphi \left(0,0,z_{4}\left(t\right)\right)-\varphi \left(0,0,z_{1}\left(t\right)\right)$$

In the case of homogeneous surfaces, one can consider $\sigma_{T}$ as a constant and by solving of Equation 7 an expression of $\sigma_{U}\left(t\right)$ is given. The current in load impedance Z is then yielded by $i\left(t\right)=i_{D}\left(t\right)=S\frac{d\sigma_{U}\left(t\right)}{dt}$ and the voltage between electrodes by $v_{Z}\left(t\right)=-Zi\left(t\right)=-\left(\varphi \left(0,0,z_{4}\left(t\right)\right)-\varphi \left(0,0,z_{1}\left(t\right)\right)\right)$ with

(Equation 8) $$\varphi \left(0,0,z_{4}\left(t\right)\right)-\varphi \left(0,0,z_{1}\left(t\right)\right)=\frac{\sigma_{U}\left(t\right)}{\pi \varepsilon_{2}}\overset{z_{4}\left(t\right)-z_{1}}{\underset{0}{\int }}f\left(z^{\text{′}}\right)dz^{\text{′}}+\frac{\sigma_{T}}{\pi \varepsilon_{2}}\overset{z_{4}\left(t\right)-z_{3}}{\underset{z_{4}\left(t\right)-z_{2}}{\int }}f\left(z^{\text{′}}\right)dz^{\text{′}}+\frac{\sigma_{T}}{\pi \varepsilon_{1}}\overset{z_{1}-z_{2}}{\underset{z_{1}-z_{3}}{\int }}f\left(z^{\text{′}}\right)dz^{\text{′}}+\frac{\sigma_{U}\left(t\right)}{\pi \varepsilon_{1}}\overset{-z_{4}\left(t\right)+z_{1}}{\underset{0}{\int }}f\left(z^{\text{′}}\right)dz^{\text{′}}$$

where $f\left(z\right)=\tan^{-1}\left(\frac{xy}{4\text{z}\sqrt{{\left(\frac{x}{2}\right)}^{2}+{\left(\frac{y}{2}\right)}^{2}+z^{2}}}\right)$.

In lateral modeling (sliding-mode) a relevant work has been carried out in (Shao, J. et al., 2020a). The authors have shown that electric field components are

$$E^{\left(X,Y,Z\right)}\left(t\right)=\frac{-\sigma_{T}}{4\pi \varepsilon \left(r\right)}\overset{a\left(t\right)}{\underset{0}{\int }}\overset{\frac{b}{2}}{\underset{\frac{-b}{2}}{\int }}\frac{\left(x-x^{\text{′}},y-y^{\text{′}},z-z_{0}^{-}\left(t\right)\right)dx^{\text{′}}dy^{\text{′}}}{{\left[{\left(x-x^{\text{′}}\right)}^{2}+{\left(y-y^{\text{′}}\right)}^{2}+{\left(z-z_{0}^{-}\left(t\right)\right)}^{2}\right]}^{3/2}}$$

$$+\frac{\sigma_{T}}{4\pi \varepsilon \left(r\right)}\overset{L+a\left(t\right)}{\underset{L}{\int }}\overset{\frac{b}{2}}{\underset{\frac{-b}{2}}{\int }}\frac{\left(x-x^{\text{′}},y-y^{\text{′}},z-z_{0}^{+}\left(t\right)\right)dx^{\text{′}}dy^{\text{′}}}{{\left[{\left(x-x^{\text{′}}\right)}^{2}+{\left(y-y^{\text{′}}\right)}^{2}+{\left(z-z_{0}^{+}\left(t\right)\right)}^{2}\right]}^{3/2}}$$

$$+\frac{\sigma_{T}}{4\pi \varepsilon \left(r\right)}\overset{a\left(t\right)}{\underset{0}{\int }}\overset{\frac{b}{2}}{\underset{\frac{-b}{2}}{\int }}\frac{\left(x-x^{\text{′}},y-y^{\text{′}},z-z_{1}\left(t\right)\right)dx^{\text{′}}dy^{\text{′}}}{{\left[{\left(x-x^{\text{′}}\right)}^{2}+{\left(y-y^{\text{′}}\right)}^{2}+{\left(z-z_{1}\left(t\right)\right)}^{2}\right]}^{3/2}}$$

$$-\frac{\sigma_{T}}{4\pi \varepsilon \left(r\right)}\overset{L+a\left(t\right)}{\underset{L}{\int }}\overset{\frac{b}{2}}{\underset{\frac{-b}{2}}{\int }}\frac{\left(x-x^{\text{′}},y-y^{\text{′}},z-z_{2}\left(t\right)\right)dx^{\text{′}}dy^{\text{′}}}{{\left[{\left(x-x^{\text{′}}\right)}^{2}+{\left(y-y^{\text{′}}\right)}^{2}+{\left(z-z_{2}\left(t\right)\right)}^{2}\right]}^{3/2}}$$

(Equation 9) $$+\frac{\sigma_{E}\left(t\right)}{4\pi \varepsilon \left(r\right)}\overset{L}{\underset{a\left(t\right)}{\int }}\overset{\frac{b}{2}}{\underset{\frac{-b}{2}}{\int }}\frac{\left(x-x^{\text{′}},y-y^{\text{′}},z-z_{1}\left(t\right)\right)dx^{\text{′}}dy^{\text{′}}}{{\left[{\left(x-x^{\text{′}}\right)}^{2}+{\left(y-y^{\text{′}}\right)}^{2}+{\left(z-z_{1}\left(t\right)\right)}^{2}\right]}^{3/2}}-\frac{\sigma_{E}\left(t\right)}{4\pi \varepsilon \left(r\right)}\overset{L}{\underset{a\left(t\right)}{\int }}\overset{\frac{b}{2}}{\underset{\frac{-b}{2}}{\int }}\frac{\left(x-x^{\text{′}},y-y^{\text{′}},z-z_{2}\left(t\right)\right)dx^{\text{′}}dy^{\text{′}}}{{\left[{\left(x-x^{\text{′}}\right)}^{2}+{\left(y-y^{\text{′}}\right)}^{2}+{\left(z-z_{2}\left(t\right)\right)}^{2}\right]}^{3/2}}$$

where L is the length of dielectrics and a(t) the relative motion distance between dielectrics (Figure 3B). During motions on overlapping surface $S_{ov}\left(t\right)=b\left(L-a\left(t\right)\right)$, a charge density $\sigma_{E}\left(t\right)$ exists, equal in magnitude with opposite sign to $\sigma_{T}$ of the material in direct contact with the electrode. On $S_{ov}\left(t\right)$ consider $-\sigma_{E}\left(t\right)$ to the top electrode and $+\sigma_{E}\left(t\right)$ to the bottom. Charge density $\sigma_{E}\left(t\right)$ is determined with the requirement that the charge density on the electrodes must be zero at any time t. Thus, on the top electrode $-\sigma_{T}a\left(t\right)-\sigma_{E}\left(t\right)$ (L−a(t))=0 is obtained. In the case where electrodes are connected to load impedance Z, $\sigma_{E}\left(t\right)$ in Equation 9 is replaced by $\sigma_{E}\left(t\right)+\sigma_{U}\left(t\right)$. However, for the moment in the Maxwell approach, no analytical general expression of $\sigma_{U}\left(t\right)$ is given and the exact solution remains challenging.

### Optimal load impedance

As shown previously, each expression of voltage depends on load impedance Z between dielectric electrodes. The choice of Z is crucial for an optimal power transfer. The optimum impedance for maximum instantaneous power is a key parameter to maximize the efficiency of TENG. Therefore, the position of optimum impedance requires to be studied for establishing a relationship with the TENG’s structural parameters and operational conditions. In this case, the TENG could be rationally controlled for applications.

In the Gauss approach (Niu et al., 2013), the authors mathematically investigated an arbitrary motion for their TENG model. When Z is neither too large (Region III) nor too small (Region I), the behavior of the TENG is in the Region II between short circuit and open circuit within which the maximum power is reached. Therefore, an optimal power transfer device should have an input impedance in the Region II. They showed that the maximum current $I_{max}$ of i(t) is given by

(Equation 10) $$I_{max}=\frac{\sigma_{T}d_{0}}{Z\varepsilon_{0}}G\left(F,y\right)$$

with $y=\frac{z_{max}}{d_{0}}$ the thickness factor and (G, F) as two functions for a cosine motion mode $z\left(t\right)=\;z_{max}\left(\frac{1}{2}-\frac{1}{2}cos\left(\frac{\pi <V>t}{z_{max}}\right)\right)$, where <V> is the average velocity over one period of motion. For their application, they used the following: maximum separation distance $z_{max}=0.001\text{m}$, the average velocity <V≥0.1m/s, the triboelectric charge density $\sigma_{T}=10\text{μC}/{\text{m}}^{2}$, and the area size of the dielectrics $S=58.0644{\text{cm}}^{2}$. The peak instantaneous power is then yielded as

(Equation 11) $$P_{max}=ZI_{max}^{2}=\frac{\sigma_{T}^{2}S<V>}{\varepsilon_{0}}F^{2}G^{2}\left(F,y\right)$$

The optimal load impedance $Z_{opt}$ satisfies the condition $\frac{\partial P_{max}}{\partial Z}$ = 0 leading to

(Equation 12) $$Z_{opt}^{gauss}\approx \frac{{\left(d_{0}+z_{max}\right)}^{2}}{S<V>\varepsilon_{0}}$$

This result can be used for estimating the optimum impedance Z of the TENG. However, the above expression is related to a specific cosine motion. The authors insisted that $Z_{opt}^{gauss}$ will somewhat deviate from this estimated value and could be used as a good reference to maximize transferred power to optimal device.

In the Maxwell approach, the authors have represented the electric model of TENG device by an open circuit voltage $V_{oc}\left(t\right)$ source in series with a time varying capacitor c(t) as shown in Figure 3C (Shao et al., 2020b).

Owing to c(t), TENG serves as an impedance to the flow of electric charges and is represented by an internal impedance $z_{T}\left(t\right)=\frac{1}{c\left(t\right)\omega }$, where ω is the angular velocity of c(t). The change in c(t) results in a change in $z_{T}\left(t\right)$ during the energy conversion process. The authors showed that $z_{T}\left(t+T\right)=z_{T}\left(t\right)$ and can be developed from a Fourier cosine series by $z_{T}\left(t\right)=z_{0}+\sum_{n=1}^{\infty }z_{n}\text{cos}\left(n\omega t\right)$. The optimal impedance is then given as

(Equation 13) $$Z_{opt}^{mawxell}=Z_{rms}\approx \sqrt{\frac{1}{T}\overset{T}{\underset{0}{\int }}z_{T}\left(t\right)dt}$$

Thus, the optimal impedance depends on the geometry and motion parameters such as frequency and maximum separation distance.

### Energy transfer device

As other sources of energy from the environment, triboelectricity is time dependent. The magnitude and frequency of the voltage peaks are primarily subjected to the mechanical force applied to the TENG, making them difficult to predict. It is therefore necessary to design an energy storage device to be able to obtain a constant and controllable power. However, the use of electric accumulators can only be considered if an energy transfer circuit is present.

This part represents one of the key points for the development of a self-powered system. Indeed, it is on this portion of the system that most of the energy produced will be dissipated.

In addition, the integration of components on textiles brings an additional constraint as the components may be subjected to multiple mechanical stresses. It will therefore be necessary to ensure that the operation of the latter is not too disturbed by the various deformations applied by the user.

Finally, the final system being intended to be autonomous, it will therefore be excluded from using active components that are externally supplied.

### Impedance matching

The internal resistance of TENGs is very high and can vary from 10^5^ to 10^7^ Ω which implies a high output voltage, but a very low current. Batteries, on the other hand, have a much lower input resistance which is generally between 10^−2^ and 10^2^ Ω.

However, the power delivered between an electrical source and a load depends strongly on their respective impedance. In fact, for the power transferred to the load to be maximum, their impedance must be adapted. It is therefore necessary to compensate for the reactive part of the source impedance as well as ensure that the resistive parts of the source and the load are equal.

It can be noted that the efficiency of the system is found to be the ratio between the power delivered to the load (therefore dissipated by its internal resistance $R_{L}$ ) and the total power supplied by the source (dissipated by the own resistance of the source $R_{S}$, as well as by $R_{L}$). We can express the efficiency *η* as a function of $R_{S}$ and $R_{L}$ as being

(Equation 14) $$\eta =\frac{1}{1+\frac{R_{S}}{R_{L}}}$$

The efficiency will be better if the load resistance is very large compared to that of the source. Here, the energy transfer should be optimal; however, this will be done despite the efficiency (as if $R_{S}=R_{L}\to \eta =0.5$).

In the case discussed here, the resistance of the TENG (the source) is much higher than that of the battery (the load), so it will be necessary to adapt the system in order to transmit a maximum of power to our accumulator.

This can be achieved by creating an L-C circuit or by using a transformer.

#### L-C circuit

The L-C circuit is generally used as a filter and also used to match the inductance of the load to that of the source. There are mainly three distinct networks: the L-C series/parallel circuit (L-circuit), the L-C circuit in π, and the L-C circuit in T.

**Figure 4:**
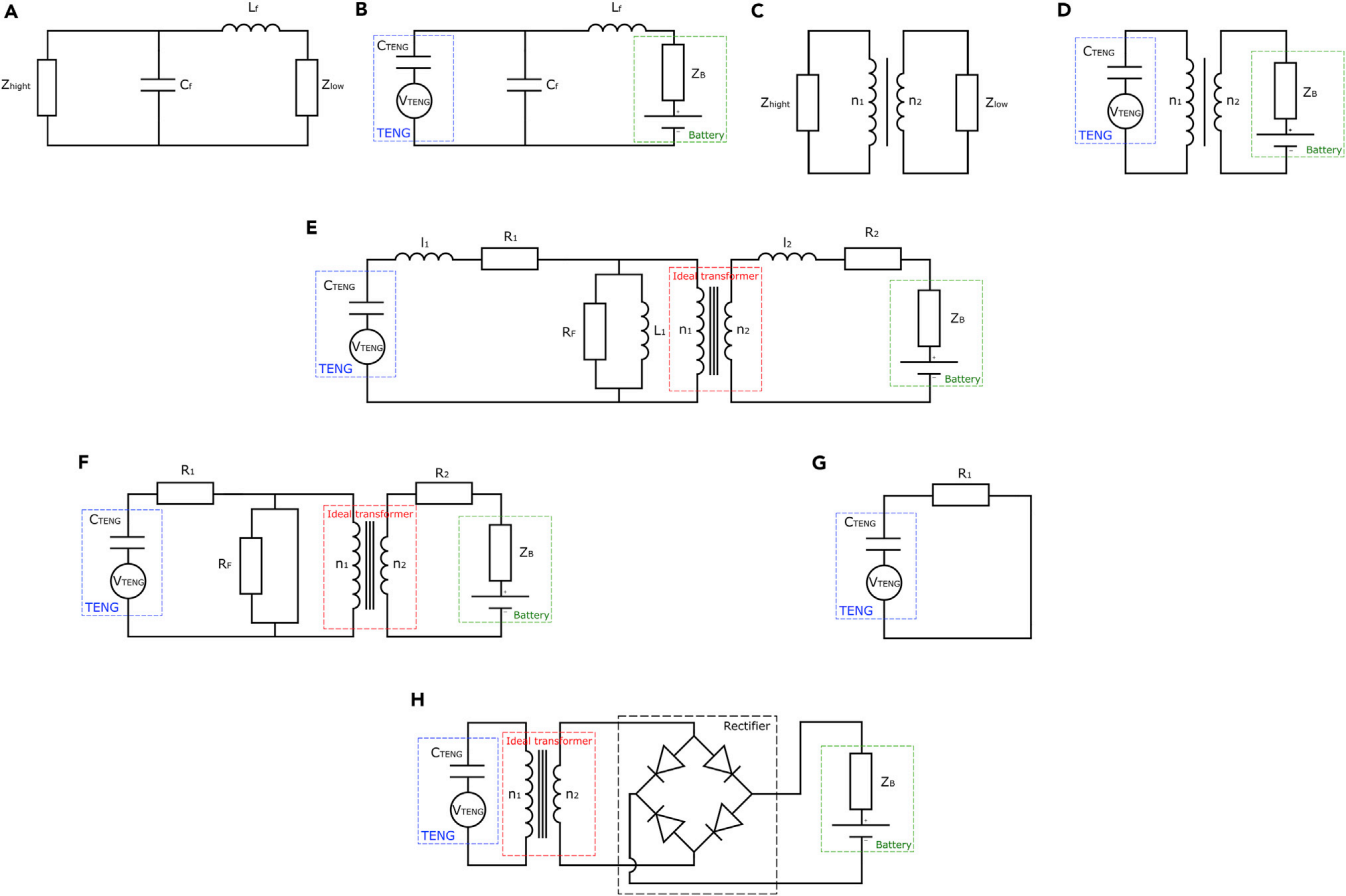
Transformer circuits for TENG (A). L-C circuit in L, (B) L-C circuit in L applied to TENG, (C) Circuit using a transformer, (D) Transformer applied to TENG, (E) Circuit using an ideal transformer, (F) Circuit using an ideal transformer in low frequency, (G) Ideal transformer applied to the TENG in low frequency simplified, (H) Circuit using an ideal transformer and a diode bridge.

The L-C circuit in π is often used when the mismatch between input and output impedance is medium or large. Circuit in T is dedicated for matching of low or medium impedance. Finally, the series/parallel L-C assembly is more suitable for high to low impedance matching. In our case, therefore, it will be necessary to promote the L-shaped circuit given in Figures 4A and 4B.The sizing of the components is then

(Equation 15) $$L_{F}=\frac{Z_{Low}}{2.\pi .f}\sqrt{\frac{Z_{High}-Z_{Low}}{Z_{Low}}}$$

(Equation 16) $$C_{F}=\frac{1}{2.\pi .f.Z_{High}}\sqrt{\frac{Z_{High}-Z_{Low}}{Z_{Low}}}$$

We can notice in Equations 15 and 16 that the components of the L-shaped circuit depend on the high impedance ($Z_{High}$ which corresponds to the TENG) and the low impedance ($Z_{Low}$ which represents the battery), as well as on the operating frequency f of the system.

This last point could be an issue as this type of adaptation is very powerful for a given frequency of operation. However, the frequency of the signal emitted by the TENG depends on the movement applied to it. It becomes then impossible to design a universal circuit as the operating frequency may greatly differ depending on the mechanical energy source. Moreover, the loss owing to the filter would be even greater in the case of a simple pulse as the majority of the frequencies contained in the signal would then be dissipated.

It will therefore be necessary to find a component with a higher bandwidth to ensure optimal energy transfer during adaptation.

#### Transformer

In order to compensate for the filter (quite selective) generated by the L-C circuits, it is possible to use transformers to adapt the TENG to charge an energy storage (a battery for example) (Figures 4C and 4D). Indeed, the transformers have the advantage to work in wide band and to be always stable (even at high rates of transformations).

In a transformer, the ratios of voltages and currents being modified between the primary and secondary, an impedance placed in the primary will not be perceived with its initial value in the secondary. This allows us to perform an adaptation by playing on the number of turns on both sides of our component ($n_{1}$ at the primary and $n_{2}$ at the secondary) via Equation 17.

(Equation 17) $$\frac{n_{1}}{n_{2}}=\sqrt{\frac{Z_{High}}{Z_{Low}}}$$

Although this element has a wide bandwidth, it is important to note that the DC (direct current) component and low frequencies will be filtered. It will therefore be possible that part of the power potentially transmitted to the battery will be strongly dissipated.

Indeed, when using a transformer, it is necessary to take into account the voltage drops in the resistance generated by the primary and secondary windings as well as their leakage inductance. The transformer can then be represented using the following equivalent diagram.

It is clear from Figure 4E that the use of a transformer at low frequency can cause a power loss via the inductance $L_{1}$.

In the case of low operational frequency of the TENG, inductance can be considered as simple wires (especially $L_{1}$ which will be very small before $R_{f}$). Therefore, the circuit presented above can be simplified as shown in Figures 4F and 4G.

In this configuration, the power generated by the TENG would not be transmitted to a storage element. Therefore, the system proposed in (Pu et al., 2016) and described in Figure 4H is not appropriate for an autonomous embedded system.

As this solution is not suitable at low frequencies, such as human motion energy harvesting for wearables, it is necessary to find another method to ensure optimal power transmission.

#### Voltage rectification and regulation of the voltage

As conventional solutions do not allow the optimal adaptation of the input impedance of the TENG with that of a storage device (for low frequency signals), several groups have developed circuits combining adaptation and tension reading.

##### Universal adaptation circuit

**Figure 5:**
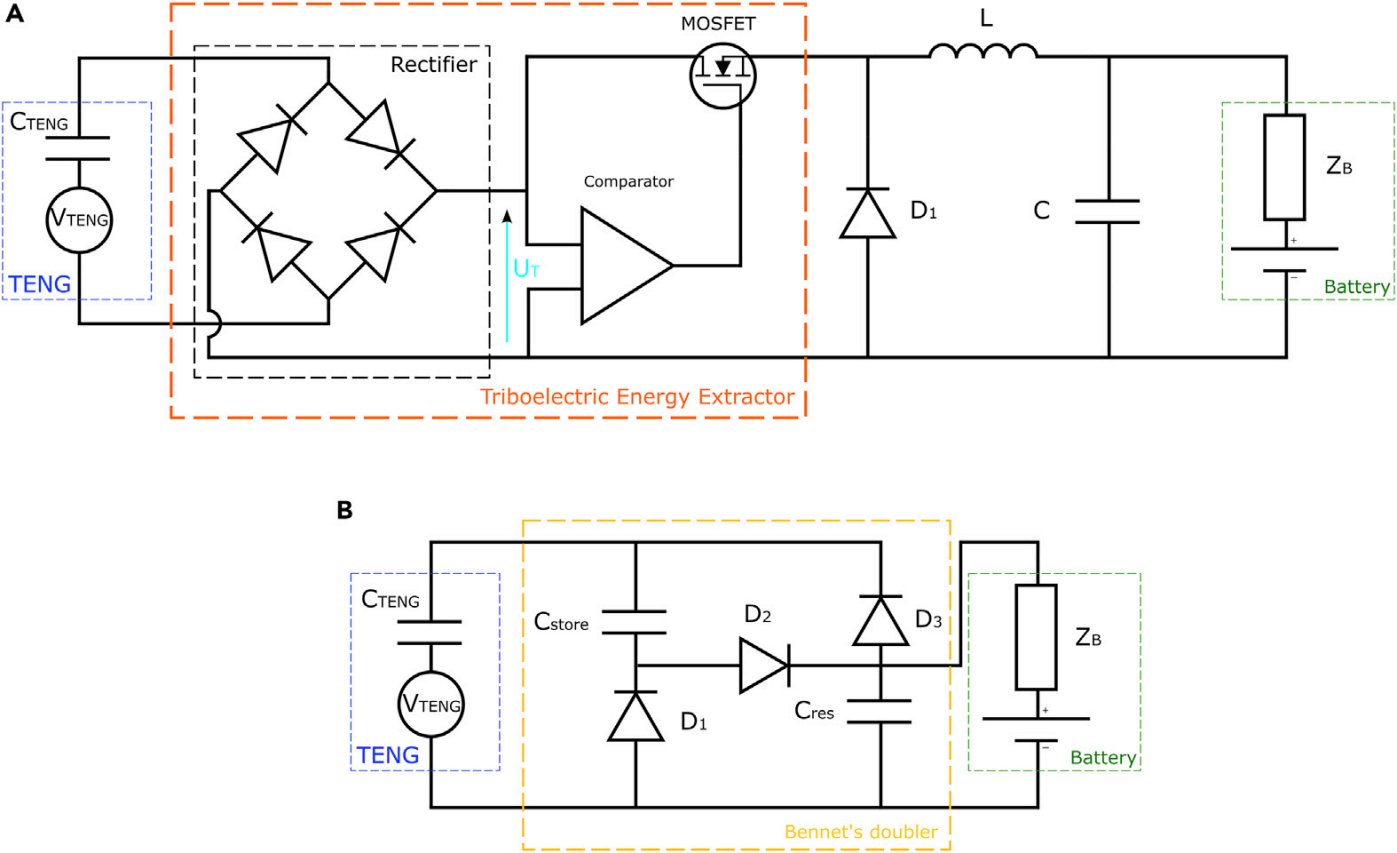
Voltage rectification ircuits for TENG (A) Universal power management circuit, (B) Circuit using a Bennet doubler.

For the circuit proposed by (Xi et al., 2017) and given in Figure 5A, an efficiency of up to 80.4% has been attained.

Here, the part in charge of energy extraction is isolated from the rest of the system as long as $U_{T}$ is lower than a threshold value $U_{ref}$ applied to one of the input of the comparator. When $U_{T}$ is large enough, the MOSFET acting as a switch is opened via the comparator. This has the effect of charging the L-C circuit. Then when $U_{T}$ becomes smaller than $U_{ref}$, the MOSFET goes to the blocking state and the L-C circuit releases the energy stored in the resistor R.

However, the threshold voltage $U_{ref}$ and the comparator must be chosen carefully as the latter must operate only with the current and voltage produced by the TENG.

##### Bennet’s doubler

The second interesting circuit for this application is the Bennet doubler, studied in (Ghaffarinejad et al., 2018) and exhibited in Figure 5B.

The topology proposed above implements a conditioning scheme represented by a rectangular charge cycle of adjustable aspect ratio. This circuit shows an exponential increase in converted energy over the operating time. Theoretically, the energy growth starts from an initial energy that is assumed to be low in the system (which is the case of the TENG).

It will be necessary to compare these two topologies in a future study in order to determine which one is the most efficient under the envisaged conditions of use, as well as to target the circuit which would be the most suitable for integration on flexible substrate.

The first two parts brought a broad overview on the physical origins of triboelectricity, and different electrical modeling of TENG as well as requirements for an optimum collection of the generated energy. The next section will focus on the implementation of TENG in textile structures.

## Textile triboelectric nanogenerators

T-TENG is a promising source of energy for wearable devices and smart textiles as it can be directly integrated into the device structure. Early in the development of TENG, textile structures have been investigated. An Increased interest in the subject is demonstrated by the spectacular growth of publications and citations doubling every year. A tremendous number of materials of choice, technologies employed and global architecture of T-TENG have been explored during this short span of time, with several new papers per week enriching this corpus (Dong et al., 2021).

The goal of this part of the review is to give a global overview on the different strategies that have been employed and developed to create T-TENGs. In order to create an efficient TENG, it is important to define what elements will be used and how they will be organized. The different data for the choice of dielectrics and conductive materials are detailed in order to have an insight into the different parameters playing a role in the performances of the device. In the case of a textile-based device, a wide diversity of techniques can be employed to fabricate the structure and the stacking of materials. Two main techniques are knitting and weaving, but both have a huge number of possible patterns, which are great tools for creativity. An overview of different possibilities explored by weaving, knitting, and other kinds of global structures is given in the second half of this part of the article.

### Choice of materials

#### Choice of dielectrics

**Figure 6:**
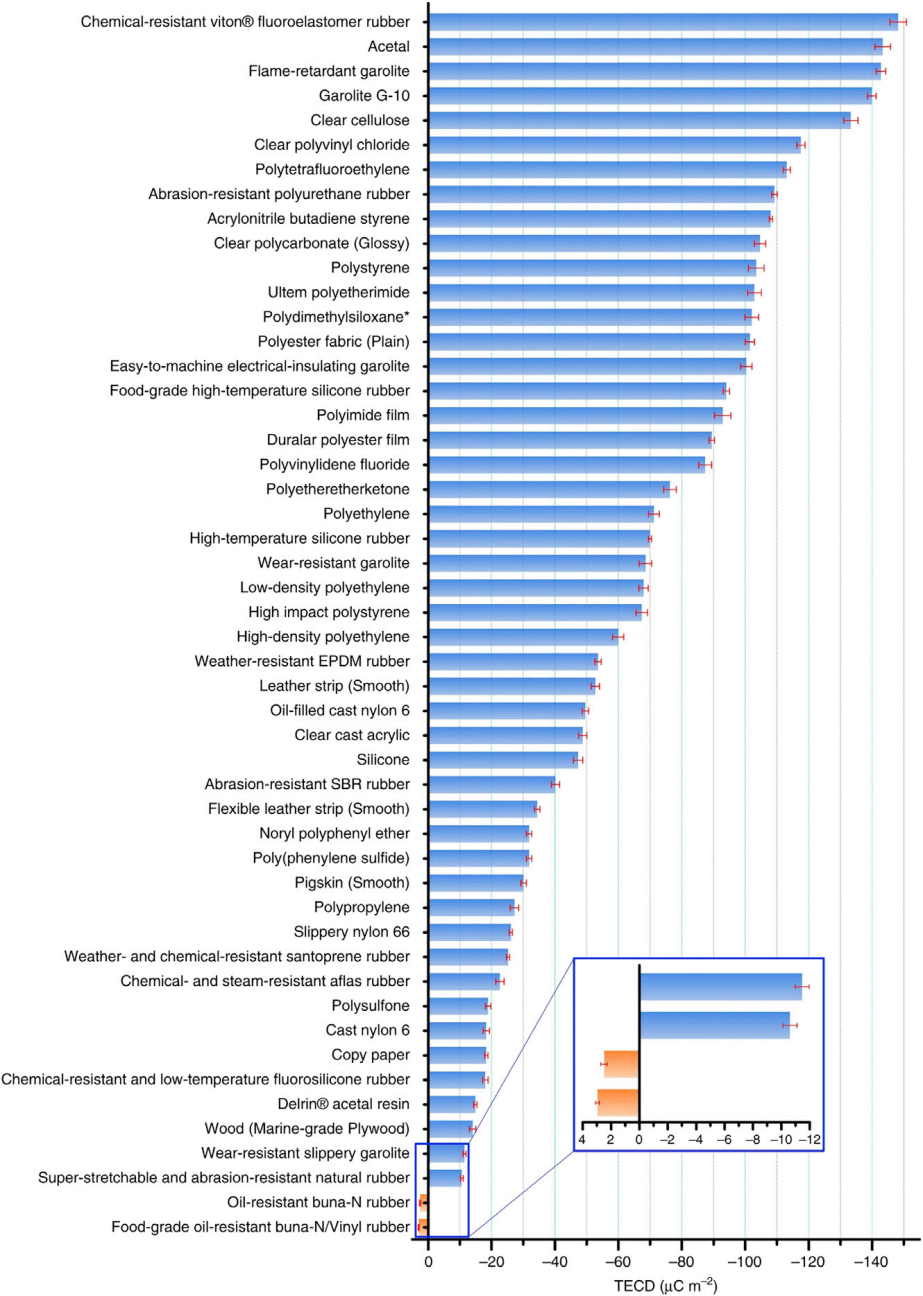
Triboelectric series. Reproduced with permission from (Zou et al., 2019) Creative Commons CC-BY license.

A triboelectric table has been long time established in an empirical way by rubbing the materials two-by-two and by classifying the materials according to their relative polarization. In the case of solid-solid contact, the effective contact surface depends on the surface state of rubbing materials, and so the surface charge density measured will depend on the surface state. In 2019, a first achievement of a methodology to define an "absolute" table has been proposed (Zou et al., 2019). In this experiment, the materials to be measured were facing a liquid mercury bath and the measurements were performed in a Faraday cage, with controlled atmosphere (nitrogen), pressure, temperature and humidity. This allowed to define the triboelectric charge densities of different materials as presented in Figure 6.

Ideally, to obtain the best performances, materials have to be put in contact in order to generate as much triboelectric surface charges as possible. To do so, the best choice is to employ materials that are as far apart as possible in the triboelectric table. In the case of textiles, the choice of available materials is more restricted: thermoplastics, natural fibers and inorganic fibers are the main categories of available yarns; and inks or coatings can enable the deposition of other specific materials on textile substrates. As detailled previously, during the contact electrification, one material will acquire a negative charge (capture of electrons) while the other one will acquire a positive charge (loss of electrons). Several chemical and physical parameters have an influence on the tendency to a certain surface to gain or lose electrons (Li et al., 2020) (Chen et al., 2020c) (Zou et al., 2020).

The presence of electron-withdrawing groups such as fluorine (–F), cyano (–CN), ester (–COOR), acyl (–CON–), carboxyl (–COOH), or nitro (–NO2) will promote the electron retention. The presence of unsaturated bonds also facilitates the acceptance of an additional electron. To do so, surface alteration via surface modification methods (such as UV–ozone treatment or plasma etching) can further increase the performance of electron-withdrawing materials.

For electron-giving materials, the electrons are weakly bound which corresponds to a low work function. Some of typical electron-donating groups are amidogen (–NH2), amide (–CONH), oxhydryl (–OH), and alkoxy (–OR), or positively charged oxygen groups (Chen et al., 2020c).

#### Choice of conductive materials

The other important element in a TENG is the conductive element allowing the circulation of electrons during the generation of energy. In non-textile TENG, the vast majority of systems rely on bulk metals, which have the advantage of mechanical resistance, excellent conductivity and reasonable price. In the case of a T-TENG, the conductivity is still of utter importance, but lightness, flexibility and breathability are other key points to improve in the design of the product in order to obtain a wearable product that keeps the advantages of a textile. Washability and mechanical resistance of the system to deformations are also important factors to consider.

In the case of bulk systems, metals are the most common conductors as they are widely available and have an excellent conductivity. The drawbacks are the heavy weight and the lack of flexibility, particularly for wearable textiles. In order to overcome them, metallic plated yarns or clothes are a good alternative as they are lighter and more compatible with textile. The lower quantity of metal causes a higher resistance, and the abrasion during washing can erode the plating which can cause a loss of performance with elapsing time. Some intrinsically conductive polymers such as PEDOT: PSS are more and more used. As they degrade at high temperature or under humidity and UV light, they are not compatible with smart textiles.

Carbon-based conductive materials are available in a wide range of techniques (filling of polymer, or different dipping/coating techniques) and inexpensive and can bring conductivity in or on insulating materials. Another possibility to bring conductivity to insulators is to fill polymers with conductive charges. Both of these techniques are not yet exploited at the industrial scale to obtain highly conductive textile but only for antistatic applications.

### Analysis of different kinds of textile triboelectric nanogenerators structures

#### Woven structures

The first T-TENG (Zhong et al., 2014) is composed of cotton coated with carbon nanotube ink yarn (CCTY), and PTFE coated CCTY. Both yarns are entangled to form a double helix, then the height of these yarn-based TENG are woven and sewn on a lab coat to form a proof of concept “power shirt”, using the energy originating from movements to power a wireless body temperature sensor system and being able to charge a 2.2 μF commercial capacitor. The maximum power density gained from this device is 0.1 μW/cm^2^ at a resistance of 80 MΩ.

Several devices have explored the design of fabric strips woven into T-TENG. In a first example (Zhou et al., 2014), strips of polyester and nylon fabric were used as dielectric layers; a silver fabric strip is pasted on dielectric layer strips as a conductive layer and then assembled to form a woven structure. Integrated into a coat, at leg joint and arm joint, it can generate energy from friction with other surfaces or with itself in deformation mode, allowing to exploit the folding movements. A similar structure sewn on cotton cloth as substrate has also been developed (Cui et al., 2015). Copper strips act as electrode and active dielectrics are nylon cloth and dacron. This system attaining a $V_{oc}$ of 2 kV has a durability tested for 5400 cycles and can stand one wash.

Woven structure based on polyester fabric strips as substrate has also been realized (Pu et al., 2015). PES is coated with nickel, then coated with parylene, to make Ni-cloth and Parylene-cloth. Once woven, the device reaches a maximum output power of 393.7 mW/m^2^ at 70 MΩ, and the energy harvested by integrating the device under a foot, under an arm or at an elbow joint. Associated to a textile-based flexible battery, a heartbeat meter with remote communication with smartphone was powered.

A traditional weaving machine was also used to create a T-TENG (Chen et al., 2018) with a technique widely available. Energy harvesting and storing parts were integrated in the same structure. As for the T-TENG, it was composed of a woven electrode, with carbon as weft and cotton as warp, and a woven dielectric textile with carbon warp and PTFE as weft. Different weaving patterns allow it to function in CS mode or in freestanding mode. As a demonstration, this device was sewn under the arm of a sweater and able to power an electric watch continuously.

Coaxial fiber made by double coating, containing an energy storing fiber at the center of the fiber (H_3_PO_4_/PVA) and TENG outside made of silicone rubber and carbon fiber, has been reported. The maximum power output of a single fiber was 1 μW at a load resistance of 100 MΩ, owing to the deformation of the fiber section. A demonstrator was designed by weaving several of these fibers and integrating them into a lab coat, under the arm. Only a low decrease in the maximum current was seen after three washes, and the system was able to power an electronic watch. These are promising results, although the large diameter of the fiber reduced the number of devices in which it can be integrated (Yang et al., 2018).

A stainless-steel yarn in silicon rubber tube as warp yarn woven with twisted yarn made of stainless steel and modified polyacrylonitrile yarn (as weft yarn) (Gong et al., 2019) has demonstrated a maximum power of 12.5 μWm^-1^ at 20 MΩ with a durability of 2000 cycles and washability of 10 cycles. It was able to power an electronic watch harvesting its energy from tensile and compressive body motion.

Liu et al. created a system based on yarn made by a conventional fabrication process and able to charge a 0.47 μF capacitor and a red light-emitting diode (LED) (Liu et al., 2019). It is composed of woven core-sheath yarn: copper wire covered by nylon and steel wire covered by polyester. The maximum output reached was 2.33 mW/m^2^ under a resistance of 50 MΩ.

One of the last woven structures is based on a single-electrode TENG yarn made from conductive polyamide yarn as a core covered with a PTFE yarn (Ma et al., 2021). The individual yarn being extremely flexible, the whole device has an excellent tailorability and wearability. The power has reached a maximum of 25 μW/m at a resistance of 1 GΩ and a frequency of 1 Hz. However, this structure is not a real power generator as it was dedicated to the detection of acid and alkali projection on personal protective equipment.

The data of the cited works about woven T-TENG are consolidated in Table 1. The best performances are reached for resistances from 50 to 100 MΩ. Coated strips have best results, but their handmade manufacturing is not compatible is not compatible with a development on an industrial scale. Within comparable design strategies, we notice that the higher the conductivity of the conductor, the higher the performance, which confirms the utter importance of this element, as well as the nature of the dielectric. These results are promising for resistance to abrasion during washing, it remains however to test the long term resistance to water and detergents, and their effect on performances.

**Table 1:** Properties of woven T-TENGs

Device Type	Triboelectric material	Electrode	Area (cm^2^)	$V_{\mathrm{oc}}$ (V)	Max Power (resistance)	Durability	Washability (times)	References
Double helix	PTFE cotton	CNT	9.1	–	1 mW/m^2^ (80 MΩ)	5 h (90 000 cycles)	–	(Zhong et al., 2014)
Taped fabric strips	Polyester nylon	Silver	432	95	–	–	–	(Zhou et al., 2014)
Taped fabric strips	Nylon Dacron	Copper	588	2000	–	5400 cycles	1	(Cui et al., 2015)
Coated fabric strips	Parylene	Nickel	50	–	393.7 mW/m^2^ (70 MΩ)	–	–	(Pu et al., 2015)
Woven yarns	PTFE cotton	Carbon	96	118	–	15 000 cycles	–	(Chen et al., 2018)
Coaxial fiber	Silicone rubber	Carbon	–	42.9	11.2 μW/m (100 MΩ)	8000 cycles	3	(Yang et al., 2018)
Core-sheath yarn	Silicone rubber-modified polyacrylonitrile yarn	Stainless steel	–		12.5 μW/m (20 MΩ)	2000 cycles	10	(Gong et al., 2019)
Core-sheath yarn	Nylon and polyester	Copper and steel	2.25	–	2.33 mW/m^2^ (50 MΩ)	12h	–	(Liu et al., 2019)
Woven fiber-based DC TENG	PTFE PA	Silver	47.6	4500	–	–	–	(Chen et al., 2020b)
Core-sheath yarn	PTFE & cotton	Conductive polyamide yarn	–	180.06 under 15 N force	605 μW under 15 N force	–	5	(Ma et al., 2021)

#### Knitted structures

One of the first examples in the literature is a wearable textile energy generator and storage based on weft-knitted TENG and supercapacitor (Dong et al., 2017b). The T-TENG is a coaxial design composed of a stainless steel/polyester blended fiber as a conductive core, coated by silicone rubber, with a maximum power density of 85 mW/m^2^. In another publication (Dong et al., 2017a), the same base triboelectric yarn is organized in 3D knitted structures. It is able to power LED systems with hand rubbing or a smartwatch with hand tapping; and the team designed also a prototype of a selfpowered dancing blanket. For a structure of 45 cm^2^, the $V_{oc}$ is 18 V and the maximum power density reached is 263.36 mW/m^2^ at 132 MΩ, proving the interest of improving the global structure in order to access to better performances.

A finer structure has been designed by Yu et al., with a core-shell structure with a stainless-steel core covered by polyurethane (Yu et al., 2017) It is the first yarn-based structure reported in the literature that can be weaved or knitted and obtained from techniques widely available in the textile industry. The drawback is that stainless steel is heavy and quite rigid, so it is not comfortable for the wearer if integrated in a garment. The maximum power is 60 mW/m^2^ at 200 MΩ, and the device was integrated underarm and in a sock to charge a smartwatch. Resistance to washing and tailorable properties were tested as it can be cut and sewn with no loss of performance.

Another core-shell structure was studied by Dong et al. based on silver-coated nylon 6 covered by braided PTFE and PA66, transformed by flat-knitting (Dong et al., 2020). The device works under stretching or compression deformation mode and can seamlessly be integrated into a traditional wear at joint. In terms of performance, $V_{oc}$ could reach 25 V and the maximum power density was 1484 μW/m^2^ at 90 MΩ with no loss of performance after 6000 cycles and 10 washes.

One last knitting example is a double face knit by plating stitch technique, with dielectric yarn on one side and silver-coated nylon 6 as a conductor on the other side (Xu et al., 2021). The system works in CS mode or coplanar sliding mode with a $V_{oc}$ of 120 V and a maximum power density of 5.58 mW/m^2^ at 70 MΩ.

These different results are grouped in Table 2. It is clearly visible that most of the knitted structures are based on core-shell yarn as the basic component. For the moment, only basic knitting has been fully exploited. As more and more complex knitting become available, especially in the fabrication of composites, a wide variety of knitted T-TENG remains to be developed.

**Table 2:** Properties of knitted T-TENGs

Device Type	Triboelectric material	Electrode	Area (cm^2^)	$V_{\mathrm{oc}}$ (V)	Max Power (resistance)	Durability	Washability (times)	References
Coated conductive yarn	PDMS	Stainless steel/polyester fiber	45	18	263.36 mW/m^2^ @132 MΩ	–	10	(Dong et al., 2017a)
Coated conductive yarn	Silicon rubber	Stainless steel/polyester fiber	–	150	85 mW/m^2^ (100 MΩ)	50,000	15	(Dong et al., 2017b)
Core-shell yarn	Spandex	Stainless steel	306	75	60 mW/m^2^ (200 MΩ)	5 h	120	(Yu et al., 2017)
Tubular knit covered yarn	PA66 and PTFE	Ag coated nylon 6 yarn	–	25	1484 μW/m^2^ (90 MΩ)	6000 cycles	10	(Dong et al., 2020)
Double face knit by plating stitch technique	PA66 PE	Ag coated nylon 6 yarn	–	120	5.58 mW/m^2^ (70 MΩ)	6000 cycles	10	(Xu et al., 2021)

#### Multilayer structures

Another category of T-TENG is composed of multilayered systems.

Seung et al. made a system based on Ag cloth coated with polydimethylsiloxane (PDMS) (Seung et al., 2015). Hydrothermal growth of ZnO nanorods before PDMS coating has increased the performance of the device that was subsequently integrated into a proof of concept for a remote access to a keyless vehicle entry system. The properties of the system were tested regarding the number of layers (from 1 to 4) and the applied force from 0.1 to 15 kgf.

Kwak et al. created a 3-layer knitted design composed of a silver middle layer (Kwak et al., 2017) surrounded by two layers composed of silver and PTFE. Different knit structures were explored; and double knitted and rib knitted revealed enhanced stretchability as they exploit compression or stretching. They delivered a $V_{oc}$ of 23.5 V and a maximum power of 60 μW at 70 MΩ.

The multilayer designed by Huang et al. is composed of conventional textile laminated with a PTFE membrane versus a knit of cotton and conductive silver-plated nylon fibers (Huang et al., 2019). As the PTFE layer can be applied to any conventional textile, it allows easy integration of this T-TENG. It reached 203 mW/m^2^ at 80 MΩ, works on freestanding lateral-sliding mode, and was integrated into a self-operating warning indicator and the powering of a smartwatch by arm swing.

These systems are particularly interesting as they have a great versatility: it is possible to tune the number of layers, the knitting/weaving pattern per layer, and the connections between them. They are a promising path for being able to design new energy generators fitting both integration-related and performance-related specifications.

Another interesting kind of layering technique is the coating of textile in order to cover yarn or fabric with a polymer, acting as a conductor or triboelectric surface.

Mule et al. designed a T-TENG based on multiple coatings (Mule et al., 2019). The conductive layer is based on a cotton cloth coated with polypyrrole (PPy). PDMS is coating is then used as the triboelectric surface. Sandpaper is used as a mold for PDMS to get surface structuration and increase performances. This system reaches an open-current voltage of 200V and a maximum power of 82 μW/m^2^ at 70 MΩ. Another exemple of T-TENG based on structured coated textile was given by Zhang et al. In this work, a commercial silver textile was coated with a mix of PDMS and carbon nanotubes by dip coating or with a brush. The brush-coated sample presented a surface structuration, which increased the generated voltage by 80% compared to traditional dip coating. (Zhang et al., 2021).

An easier comparison of these results is possible with Table 3. The different structures have great disparities in performance, and as the performances are not always per surface, the comparison is not possible. It is nevertheless possible to note the great durability of the different samples. Unfortunately, the resistance to washing was not systematically tested, as delamination can occur and annihilate the bonding of coating, or integrity of multilayered structure can be destroyed by mechanical constraints.

**Table 3:** Properties of multilayered, coated, and non-conventional T-TENGs in the literature

Device Type	Triboelectric material	Electrode	Structure	Area (cm^2^)	$V_{\mathrm{oc}}$ (V)	Max Power (resistance)	Durability	Washability (times)	References
Hydrothermal growth + coating	PDMS	Ag	Multilayer	–	120	1.1 mW (1 MΩ)	12000 cycles	–	(Seung et al., 2015)
Knitted fabrics	PTFE	Ag	Multilayer	100	23.5	60 μW (5MΩ)	–	–	(Kwak et al., 2017)
Core-shearth yarn	Silicon rubber	Conductive Nylon yarn	Yarn		20	11 W/m^3^ (compression)	50000 cycles	10	(Dong et al., 2018)
Conv textile PTFE laminated vs. cotton + conductor	PTFE/cotton	Conductive silver-plated nylon fibers	Multilayer	–	400	203 mW/m^2^ (80 MΩ)	500 cycles	1	(Huang et al., 2019)
Coated conventional textile	PDMS	Polypyrrole-coated cotton	Coated textile	–	200	82 μW/m^2^ (70 MΩ)	5000 cycles	–	(Mule et al., 2019)
Woven fiber-based DC TENG	PTFE PA	Silver	Woven	47.6	4500	–	–	–	(Chen et al., 2020b)
TENG-supercapacitor hybrid yarn	PTFE	Conductive carbon cloth yarn	Textile array	–	400	–	1000 cycles	1	(Mao et al., 2021)
Coated yarn	PEDOT/Al foil	Ni coating	–	–	0.45	1.1 mW (30 kΩ)	6000 bendings 3350 slidings	–	(Meng et al., 2021)
Coated textile	PDMS + CNT vs. nylon	Silver textile	–	9	51.2	99.8 mW/m^2^ (200 MΩ)	10000 cycles	–	(Zhang et al., 2021)
Tubular structure of opposite Poisson ratio materials	PVC/PDMS	Stainless steel	Yarn integrated on textile	–	46	52.36 mW/m^2^ @ 10 MΩ	10000 cycles	–	(Guan et al., 2022)

#### Other structures of interest for bending movement energy harvesting

A silicone rubber core in a hollow fiber application (Dong et al., 2018) was developed for integration in smartglove and more generally for gesture recognition, and its integration as a warp for large-sized weaving was validated.

Mao et al. proposed an original core-shell structure (Mao et al., 2021) with a core made of a conductive carbon cloth yarn coated with PDMS/MnO_2_, and the shell being a PTFE yarn warped around it. This system harvests energy from compression while integrated into a textile array.

An original tubular structure based on opposite Poisson-ratio materials (Guan et al., 2022) TENG device could harvest energy from stretching movements. When extended, the outside stainless steel braided sleeve shrinks and the inside tube made of PDMS coated stainless-steel yarn expands, which allows the integration at joints, as was proven by the demonstration of a gesture recognition glove in the publication.

Although the structures described in this section are bulkier than woven or knitted structures, they offer the possibility to exploit compression and folding areas as energy-generating resources.

As the first T-TENG was reported in 2014, a wide diversity of textile materials, structures, and design have been explored so as to adapt TENG to most of the movements that can be harvested to produce energy. The evolution of the designs employed also shows that the demonstrators are more and more realized with techniques compatible with the textile technologies used on an industrial scale, which paves the way toward the realization of T-TENG widely available. A new generation of T-TENG is now emerging (Chen et al., 2020b; Meng et al., 2021) producing oscillating positive current. This may lead to a leaner design and more efficient energy management circuit. As a result, less components are needed, paving the way for the integration of the electronic circuit into textiles. Nevertheless, the seamless integration of the complete system on textiles, including the energy transfer circuit, is difficult and has not yet been achieved. This integration requires a good understanding and control of the different processes and technologies available to interconnect the electronic components of textiles including T-TENGs.

## Electronics system integration on textiles

In this section, an overview of the design of a complete tribogenerator system and integration of the energy transfer circuit onto textiles is discussed. To the best of our knowledge, the current literature covers little of this area. A recent example (Cong et al., 2020) shows the integration into the textile of a diode bridge component acting as a transfer circuit. From the literature seen in the previous sections, more complex circuits may be required for the energy conversion of a triboelectric generator and it is then necessary to identify which heterogeneous integration techniques are currently available for their incorporation of textiles. Although methods for integrating rigid PCB-based electronic circuits on textiles have been proposed in the literature, such as embroidery (Coyle et al., 2009) or screwed force-fit connection (Simon et al., 2011), the resulting prototypes seem bulky and uncomfortable. Therefore, only the direct integration of components or at least flexible PCB will be reviewed.

In the following, we will focus on two different building blocks essential for the realization of textile electronics:

1. Achieving conductive tracks on textile: they must have a low electrical resistance as well as dimensions compatible with the components mounted on the surface of the electronics (minimum pitch = line width + spacing = 400 μm)
2. Integration of electronics on textile: the circuit can be realized outside the textile on a flexible substrate but preferentially directly on the textile substrate with the help of conductive tracks and the discrete integration of the components on the textile tracks.

### Conductive tracks for textile applications

Conductive tracks on textiles are a fundamental building block for the realization of electronic circuits on textile fabrics. They must have appropriate electrical conductivity, lateral resolution and withstand mechanical stresses and washing constraints. In addition, they must allow the interconnection of various electronic components such as passive components, sensors, microprocessors, batteries, and so forth.

Conductive tracks can be embedded during the manufacturing of the textile or carried out on the final fabrics using various techniques. This section will give an overview of the different processes available to achieve reliable flexible circuits on textiles.

#### Conductive tracks embedded during manufacturing of the textile

**Figure 7:**
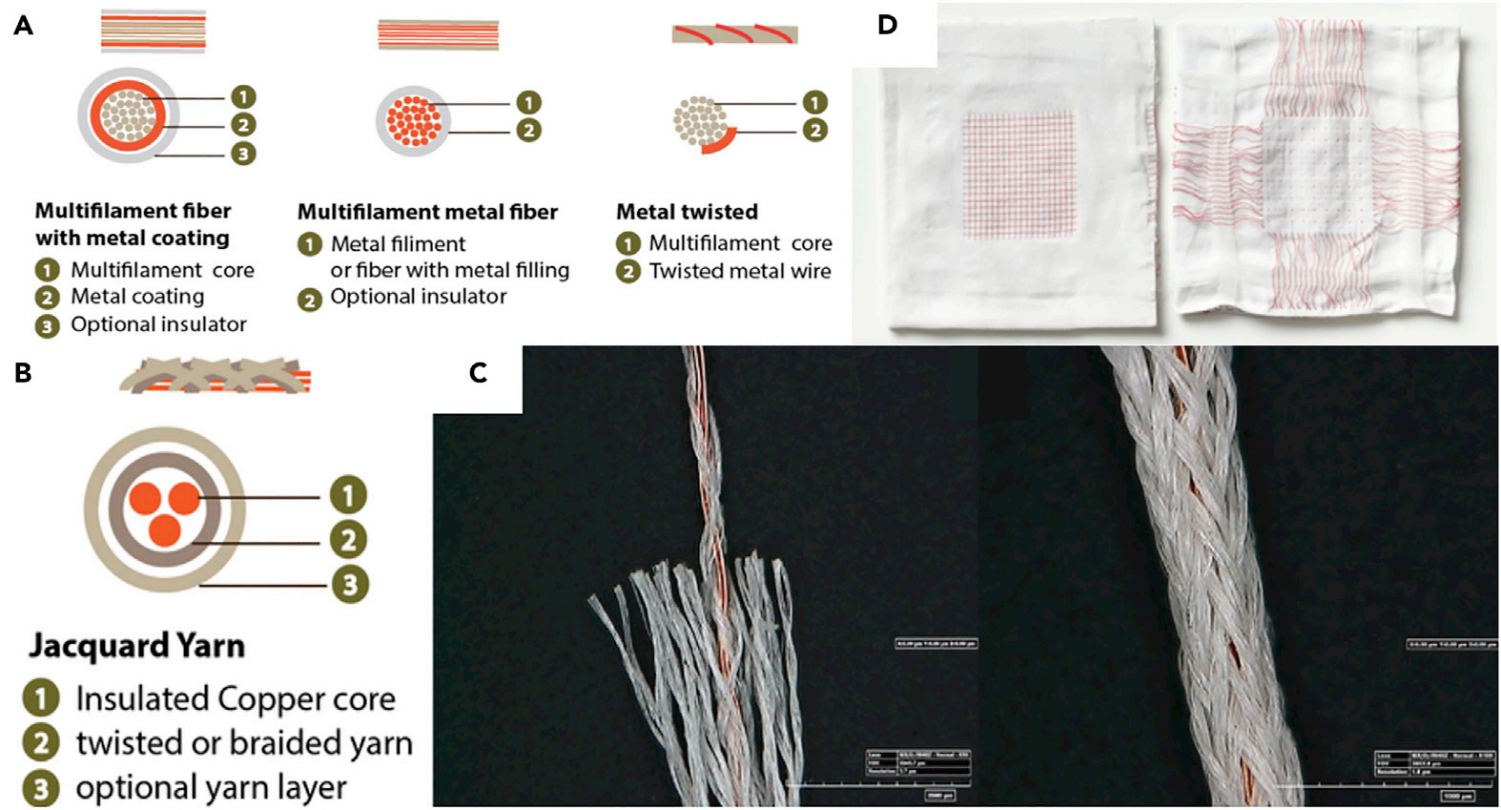
Structures and integration of textile conductive yarns (A) Conductive yarn structures: multi-filament fiber with metal coating, multi-filament metal fiber, metal twisted on multi-filament core. Reproduced with permission from (Poupyrev et al., 2016). Copyright 2015, The Royal Society of Chemistry. (B) Jacquard yarn structure. Reproduced with permission from (Poupyrev et al., 2016). Copyright 2015, The Royal Society of Chemistry. (C) Jacquard yarn micrographs.Reproduced with permission from (Poupyrev et al., 2016). Copyright 2015, The Royal Society of Chemistry. (D) Textile integrating Jacquard yarn. Reproduced with permission from (Poupyrev et al., 2016). Copyright 2015, The Royal Society of Chemistry.

Conductive threads can be knitted or woven during the textile manufacturing process. They must have the appropriate mechanical properties to be compatible with the fabrication of the textile, i.e., low rigidity, low friction, and acceptable mechanical strength. Therefore, conventional metallic wires used in electrical cables cannot be used alone as they cannot be properly processed during textile fabrication. Furthermore, the insertion of these conductive yarns must also preserve the inherent properties of the textile, that is to say: breathability, flexibility, and eventually stretchability; while also exhibiting the lowest electrical resistivity to be compatible with both the DC and AC requirements of electronic circuits. To meet all these requirements, different strategies can be considered to intimately incorporate metal in a textile yarn. These main conductive textile structures are illustrated in Figure 7A. On one hand, metal-coated polymer filament is among the most commercially available conductive yarn; on the other hand, a metallic filament may be used. It may also be twisted around a polymer yarn (Stoppa and Chiolerio, 2014) (Ojstršek et al., 2021). Optionally, a layer of polyurethane (PU) can be wound up for insulation or protection purposes.Silver and copper are mainly used owing to their excellent electrical properties. Silver suffers from low-stress resistance and washability (Paradiso et al., 2014). Cu alloys allow easy soldering/interconnection of components (Poupyrev et al., 2016). A wire grid has been demonstrated on a woven fabric incorporating copper wires. The 40 μm diameter copper wire was PU coated and separated by 0.57 mm. The linear DC resistance was around 15 Ω/m and the transmission lines operated up to 2 GHz (Locher and Troster, 2007). Stretchable fabric printed circuit board (FCB) has been tested on various woven textiles using polyamide yarns coated with silver nanoparticles. These conductive wires are flexible and robust. The DC resistance was about 200 Ω/m. The “⅓ twill weave design” guarantees the lowest electrical variation (5%) under a mechanical stress of 30% (Li et al., 2019).

Within the framework of the Jacquard project, a new conductive yarn has been developed with mechanical properties comparable to textile fibers. It consists of twisted strands of copper threads braided with silk fibers. Here again, a layer of PU is overbraided for protection against external stresses (Poupyrev et al., 2016). The structure and integration of the developed yarn are illustrated in Figures 7B, 7C and 7D. Gaubert et al. tested conductive threads (silver and stainless steel) seamlessly knitted on cotton fabric to develop sensors to prevent bedwetting. The linear DC resistance was <600 Ω/m for silver and between 2500 and 4000 Ω/m for stainless steel (Gaubert et al., 2020). To monitor human locomotion, two types of conductive yarns were tested: a mixture of polyester and stainless-steel spun yarns (350 Ω/m) and yarns made up of twisted filaments covered with silver (Catarino et al., 2013). Paiva et al. used silver-coated polyamide fibers knitted on a t-shirt for EMG and ECG purposes (Paiva et al., 2019).

Complementary to the insertion of conductive fibers during the fabric process, conductive tracks can be performed on the finished textile using different techniques such as embroidery, printing, patterning of deposited conductive layer, and lamination of a conductive pattern on the textile.

#### Conductive tracks done on the finished textile

##### Embroidery

**Figure 8:**
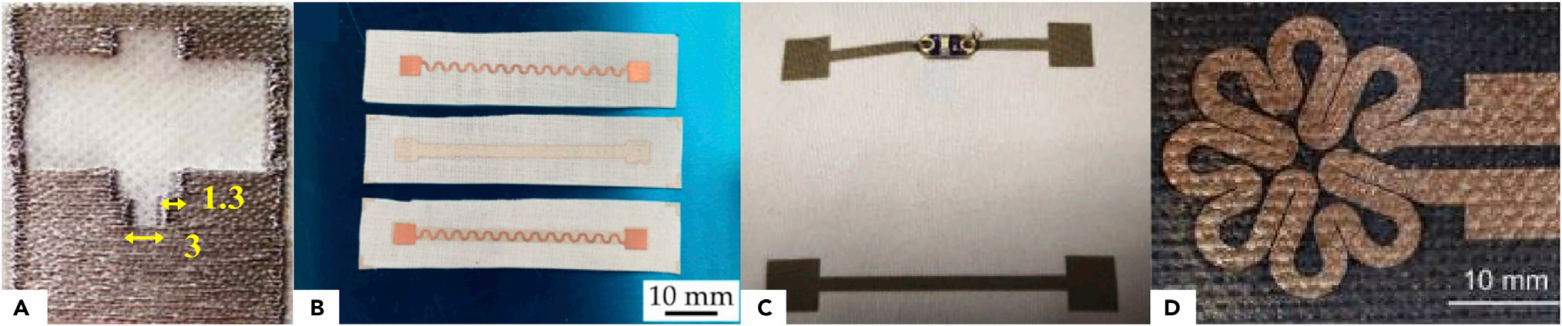
Textile conductive tracks realized with different processes (A) Antenna embroidered on textile. Reproduced with permission from (Kiourti and Volakis, 2015). Copyright 2014, IEEE. (B) Ag layer screen printed on a pre-patterned Cu serpentine. Reproduced with permission from (Koshi et al., 2020a). Creative Commons CC-BY license. (C) Inkjet-printed conductive pattern on textile. Reproduced with permission from (Kim et al., 2019). Copyright 2018, WILEY. (D) Patterned Au metallic layer, obtained with PVD process, on textile substrate Reproduced with permission from (Pawlak et al., 2018). Copyright 2018, Emerald.

Embroidery utilizes the same kind of conductive yarns as previously discussed. Again, copper, silver, or a combination of both are mostly used. Rotzler et al. performed a comparative study of the effect of washing cycles on different kinds of conductive tracks. The embroidery tracks were aramid multifilament wrapped with Cu/Ag wires. They concluded that the washability performance does not only depend on the type of conductive tracks but also on the used fabric structure (Rotzler et al., 2020). An NFC circuit was developed using conductive yarns made of copper fibers twisted on a PET thread embroidered on cotton fabric (Garnier et al., 2021). Figure 8A depicts a 2.4 GHz antenna that has been fabricated by embroidering Cu (inner) and Ag (outer) coated fibers on a textile. Using thinner fibers and increasing the embroidery density, the measured surface conductivity was close to the copper one with an improved geometrical resolution of about 0.3 mm. This kind of yarn is flexible and exhibited excellent mechanical properties (Kiourti and Volakis, 2015).

Embroidery allows the fabrication of conductive tracks with good conductivity and mechanical robustness but suffers from poor lateral resolution, low throughput, and high manufacturing cost. Consequently, additive printing techniques such as screen printing and inkjet printing are very attractive because screen printing allows high throughput, while inkjet printing is a versatile technique. Both organic and inorganic inks can be used; but to achieve high conductivity, metallic inks are favorable.

##### Screen printing

Screen printing is a well-established technique that allows the deposition of a patterned thick conductive layer of a few microns through a stencil mask. Silver and silver composite inks are mainly used because of their excellent conductivity and resistance to oxidation. Composite inks made of silver and polymers (fluoroeslastomer or PU) have been developed to improve the stretchability of the printed layer (Jin et al., 2017), (Hong et al., 2019). The electrical and mechanical performances are strongly dependent on the textile structure. Better lateral resolution and resistivity have been observed on textiles with lower surface pore (Hong et al., 2019); while the influence of penetration of the ink on mechanical robustness (Koshi et al., 2019) and ease of component interconnection (Jin et al., 2017) have been demonstrated. Interestingly, Koshi et al. have screen printed a silver layer on a pre-patterned copper serpentine, illustrated in Figure 8B, to improve the mechanical robustness (Koshi et al., 2020a).

Using screen printing, lateral resolution down to 200 μm (Kim et al., 2009) and sheet resistance down to 0.06 Ω/□ have been obtained (Jin et al., 2017). To improve mechanical robustness and resistance to washing, a PU passivation layer can be laminated on top (Karaguzel et al., 2009).

##### Inkjet printing

Inkjet printing is a contactless and maskless technique that can be used to deposit desired conductive patterns in the right place. Consequently, this technique allows the rapid test of different designs without the need of fabricating expensive masks and requires very little amount of materials (Karim et al., 2017), (Stempien et al., 2018). However, inks have low viscosity which induces undesirable ink spreading both in-plane and through the fabric (Shahariar et al., 2019). Moreover, numerous passes are required to achieve an acceptable thickness layer and conductivity (Kim et al., 2019). Spreading effects can be minimized by modifying the textile surface (chemical surface treatment or interface layer) (Krykpayev et al., 2017), (Nechyporchuk et al., 2017), (Karim et al., 2019) and by selecting the proper textile structure (Kim et al., 2019). Dense structure with small pores will reduce the ink spreading (Karaguzel et al., 2009), (Zhang et al., 2018). Lateral resolution down to 50 μm can be achieved with excellent electrical properties, but the interconnection of components remains an issue owing to the printed layer’s small thickness (around one micron). An example of inkjet printed e-textile is given in Figure 8C (Kim et al., 2019).

##### Laser ablation

Laser ablation of a conductive layer deposited by plasma vapor deposition (PVD) has also been tested by several groups. Pawlak et al. have tested the laser ablation of a gold PVD layer (300 nm) deposited on four composite textiles and also the patterning of the Ag/Au layer deposited on a plasma-pretreated Cordura textile. The resulting pattern can be seen in Figure 8D. Composite textile exhibits a polymeric top membrane (PU, PTFE, and so forth) which lowers the roughness of the fabric surface (Pawlak et al., 2017, 2018). Stempien et al. tested the laser ablation of Ag and Cu layers (Stempien et al., 2016). More rarely, patterning of a PVD layer can also be performed through a shadow mask (Kim et al., 2009).

Finally, conductive foils or textiles can be patterned by laser ablation and then transferred or laminated onto the textile. Conductive fabrics can either be made of conductive fibers or consist of a thin metal layer deposited on top of the textile surface. Laser-structured Ni/Cu and Sn/Cu plated textile have been ironed onto the fabric without affecting significantly the properties of the textile (Mehmood et al., 2019), (Buechley and Eisenberg, 2009). Rotzler et al. investigated both the lamination of copper meander foil embedded in TPU and a laser-structured silver nylon textile (Shieldex) (Rotzler et al., 2020). Silver conductive textiles patterned by laser ablation have been widely studied (Dils et al., 2019), (Escobedo et al., 2020). The combination of laser cut copper foil with the screen printing of a silver paste has also been evaluated (Koshi et al., 2020a). Lateral resolution of these conductive tracks is related to the characteristics of the used laser and can be as low as 100 μm. The resistivity is quite good and mainly depends on the choice of the metal and thickness of the layer. The interconnection of electronic components either by soldering process or using conductive paste is relatively easy and robust.

Textile conductive tracks can be obtained by different processes. The main ones are synthetized and compared in Table 4. According to the final application, one process may be preferred to the others. For example, if a high resolution is required to integrate small components, inkjet printing should be preferred as its finest resolution is one order of magnitude lower than the other processes. Similarly, if the conductive tracks require a good mechanical resistance, conductive composites obtained through inkjet or screen printing should be considered. They also exhibit low electrical resistance suitable for low-impedance applications.

**Table 4:** Processes reported for achieving conductive tracks on textile

Process	Electrical performance	Lateral resolution (μm)	Thickness (μm)	Mechanical behavior	Material	References
Woven or knitted fibers	15.7 Ω/m (warp) 17.2 Ω/m (weft)	300	40±8 μm diameter yarn	–	Copper wire	(Locher and Troster, 2007)
Embroidery	0.8–6.7 Ω/m	300	–	–	Copper and Silver coated yarn	(Kiourti and Volakis, 2015)
Screen printing	0.06 Ω/□	200	1–10	Elongation at break 215–450%	Silver fluoro-elastomer	(Jin et al., 2017)
Inkjet printing	0.03–4.7 Ω/□	50–100	0.3–1	More than 1000 bending cycles	Graphene - Silver composite	(Karim et al., 2019)

### Heterogeneous integration of electronics onto textiles

The use of silicon-based components ensures functionality on par with aimed applications. Numerous research studies have been carried out on circuit board and component integration onto textiles. On the one hand, circuit boards have the advantage of employing mature packing processes that provide reliable electrical connections of electronic components and mechanical support to the circuits. On the other hand, this reliability is achieved with rigid board (FR4 epoxy substrate) at the expense of the textile properties such as extensibility and conformability. Therefore, in this review, only flexible substrates (plastic substrate, woven textile, and so forth) will be presented as the TENG system is supposed to be worn and expected to be comfortable.

In the literature, several processes compatible with this objective have been identified. Among them, adhesive bonding and soldering will be more discussed. The main challenge is to create an interconnection between the metallic leads or other connectors of integrated circuits with the conductive textile materials. Such interconnections have to show good mechanical and electrical properties in order to attach the component to textile and also power the components or transmit the signal.

#### Integration of flexible circuits

**Figure 9:**
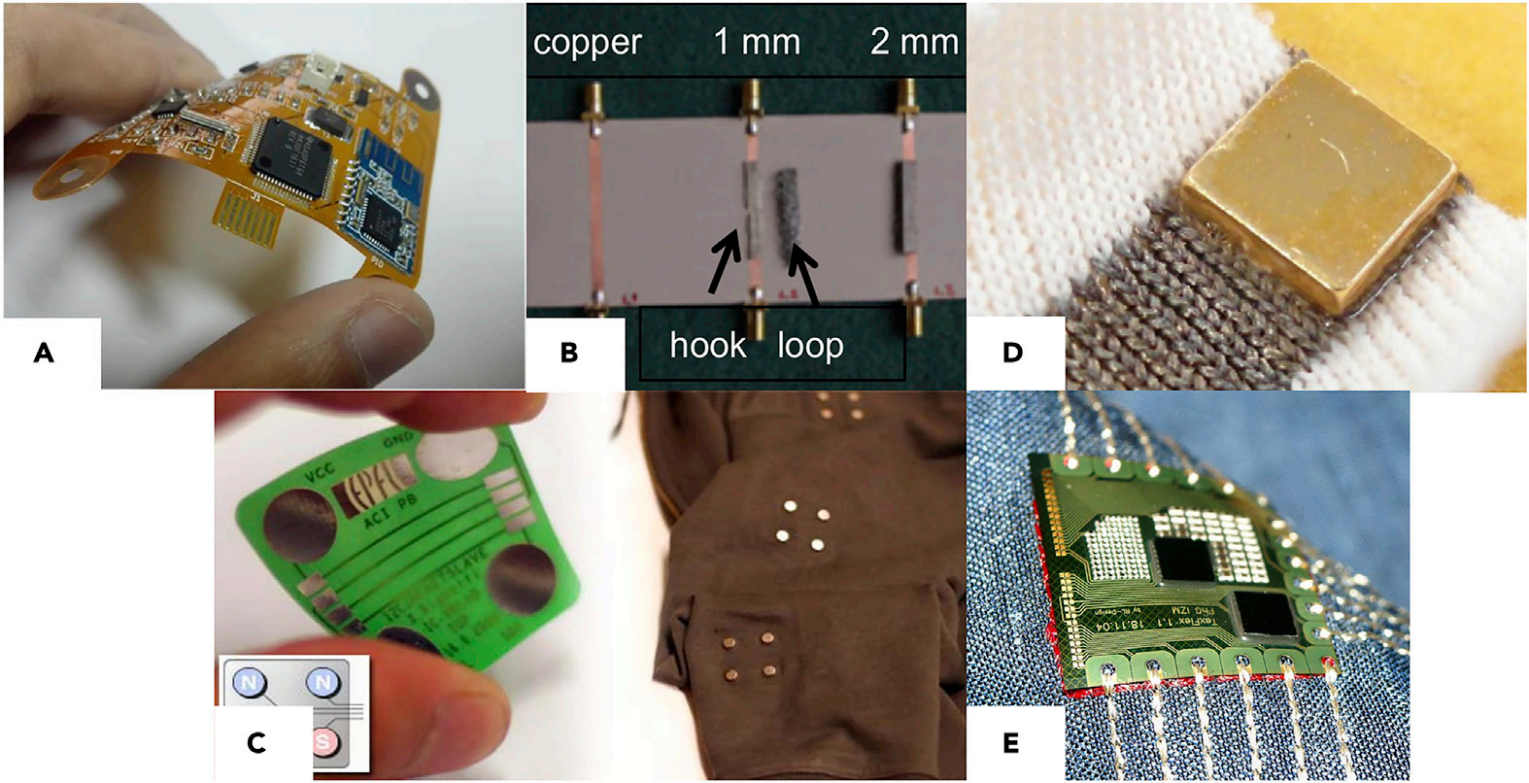
Different types of interconnections between flexible electronics and textile conductive tracks (A) FPCB-based ECG module, incorporating snap fasteners, under bent state. Reproduced with permission from (Cai et al., 2017). Copyright 2017, IOS Press. (B) Test jig of hook and loop connectors for textile wireless systems. Reproduced with permission from (Seager et al., 2013). Copyright 2013, WILEY. (C) Magnetic connectors on a flexible substrate to be connected on a jacket. Reproduced with permission from (Righetti and Thalmann, 2010). Copyright 2010, IEEE. (D) Magnet bonded with adhesive on a textile conductive substrate. Reproduced with permission from (Scheulen et al., 2013). Copyright 2013, ACM. (E) Flexible electronic module connected with conductive yarn by embroidery. Reproduced with permission from (Linz et al., 2005a, 2005b). Copyright 2005, IEEE.

Traditionally, the textile substrate and the electronics are obtained by separate processes. Hence, the need for ways to attach the electronic module afterward. The attachment can be permanent, but in some cases, a removable one could be more suitable. Indeed, the washing process of a garment involves mechanical and chemical constraints than can deeply damage the electronic module if it is not strongly protected. Snap fasteners are a way to obtain such removable interconnection as they are made from metal and already massively used by the textile industry. The use of snap fasteners has been reported, see Figure 9A, to connect a flexible printed circuit board (FPCB)-based ECG processing module to a smart shirt (Cai et al., 2017). Following the same logic of modular interconnects, one may use a conductive hook and loop fastener as an interconnection for smart textiles. Hook and loop fasteners are commonly used in garments, and conductive hook and loop are commercially available (“Electromagnetic Field Shielding Fabrics, 2021). They have been investigated for RF application (Seager et al., 2013) as shown in Figure 9B. This kind of interconnection provided a sheet resistance of 1.8 Ω/□ for the hook and 1.4 Ω/□ for the loop, leading to an expected contact resistance of 0.8 Ω for a 1-inch-wide connection. Flexible connectors for RF applications have been used at low microwave frequencies. In order to extend the frequency range of these connectors, higher conductivity is needed which may be envisioned through the electroplating of the hook and loop connector. However, the process stiffens the connector which may not remain suitable for flexible applications.

Another modular interconnection available in the literature is about the use of neodymium magnets as a mechanical and electrical linker (Righetti and Thalmann, 2010). As presented in Figure 9C, magnets can be embedded in both flexible circuit and garments to ensure the mechanical link between them. As magnetic materials show high conductivity, they may also be used as electrical contacts (Scheulen et al., 2013). As shown in Figure 9D, magnets can be connected using conductive paste onto a conductive textile. Obtained results for a contact area of 8 mm^2^ show a low contact resistance (from 1.9 to 3.7 Ω) with a nickel-coated magnet. Gold-coated magnets exhibit lower resistances (from 0.3 to 0.8 Ω) in the same conditions. Mechanical testing has been performed on the adhesive joints showing a maximum bearable force between 24 and 35 N for 8 mm^2^ contact area. Another point highlighted by the authors is the selfaligning properties of the magnets. One may envision circuit autoalignment by using magnets as interconnections.

Similarly, as wearable rigid printed circuit board (PCB), such as Lilypad Arduino which were designed to be embroidered on textile, embroidery can also be considered to integrate (FPCB), as illustrated in Figure 9E.

Different processes enable the integration of an FPCB on textile. The main ones are synthetized and compared in Table 5. Although the snap fasteners may be twice the size of the other interconnections, their contact resistance is one order of magnitude lower. Despite mechanical reliability has not been investigated in correlation with their electrical behavior, one may expect great mechanical robustness owing to the common use of snap fastener in garments. For a smaller integration, one should consider to sew/embroider the FPCB onto the textile substrate as it exhibits the smallest contact width. Contact resistance achieved with embroidering is higher than snap fastener but still in the same order of magnitude as the remaining processes.

**Table 5:** Processes reported for attaching FPCB on textile

Process	Contact resistance (Ω)	Contact width (mm)	References
Snap fastener	0.05	5–8	(Cai et al., 2017)
Hook and loop	0.8	3.5	(Seager et al., 2013)
Magnets	0.3–0.8	5	(Righetti and Thalmann, 2010)
Embroidering	0.395	2	(Linz et al., 2008, 2012a)

Linz et al. have extensively investigated the embroidering process and characterized the electrical connection (Linz et al., 2005a, 2005b, 2008). They even proposed a theoretical model of the resulting embroidered contact (Linz et al., 2010, 2012a). The conductive yarn, silver-coated polyamide—namely Statex 117/17 twine produced by Shieldex, was embroidered with semiprofessional (Bernina artista 200) and professional (ZSK JCZ 01) embroidery machines. The flexible circuit board was 50 μm polyamide foil structured on both sides with 17 μm copper, 5 μm nickel, flash gold, and 15 μm solder resist.

In this preliminary section, methods to integrate complete electronic circuits directly on the textile in a permanent or temporary manner have been addressed. This enables to preserve the high computational power offered by electronics as well as keeping their conventional fabrication process, which is reliable and efficient at the industrial scale.

Nonetheless, the electronic substrate, either rigid or flexible PCB, impaired the wearability of a garment by reducing its breathability, drapability, and even aesthetics. Hence, there is the need to go a step further by integrating directly the components onto the textile substrate.

#### Integration of components

**Figure 10:**
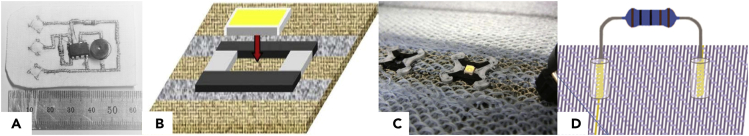
Different ways of integrating components directly onto textile conductive tracks (A) Embroidered circuit. Reproduced with permission from (Briedis et al., 2017). Copyright 2017, Elsevier Ltd. (B) Design of a 3D printed LED holder. Reproduced with permission from (Grimmelsmann et al., 2016). Copyright 2016, Elsevier Ltd. (C) Lit LED, contacted by conductive 3D printing filament (black) to conductive Shieldex yarn (silvery) Reproduced with permission from (Grimmelsmann et al., 2016). Copyright 2016, Elsevier Ltd. (D) Insert connector for detachable connection. Reproduced with permission from (Li et al., 2019). Creative Commons CC-BY license.

As illustrated in Figure 10A, Briedis et al. have tried to embroider the components despite the limited precision of this process (finest pitch reported of 0.75 mm embroidery needle diameter and 1 mm minimum embroidery machine step shift) (Briedis et al., 2017). Nonetheless, it should be noted that to realize the complete electronic circuit integrating electronic components, their leads have been manually passed through the fabric and then folded before the machine cycle was resumed.

Although the integration of surface-mounted components cannot be considered with embroidery, an integration method of surface-mounted devices (SMD) on conductive textile substrate, based on 3D printing, have been proposed (Grimmelsmann et al., 2016). Figures 10B and 10C illustrate a 3D printed LED holder that was printed on a textile substrate integrating conductive yarns with both conductive and nonconductive PLA to ensure mechanical and electrical interconnections, respectively. Then a surface-mounted LED was placed inside the holder. If a detachable connection is needed, a solution may consist in using insert-connectors as presented in Figure 10D (Li et al., 2019). The circular connector hole is made with a conductive yarn forming a tube that is further embedded into PDMS.

##### Adhesive bonding

**Figure 11:**
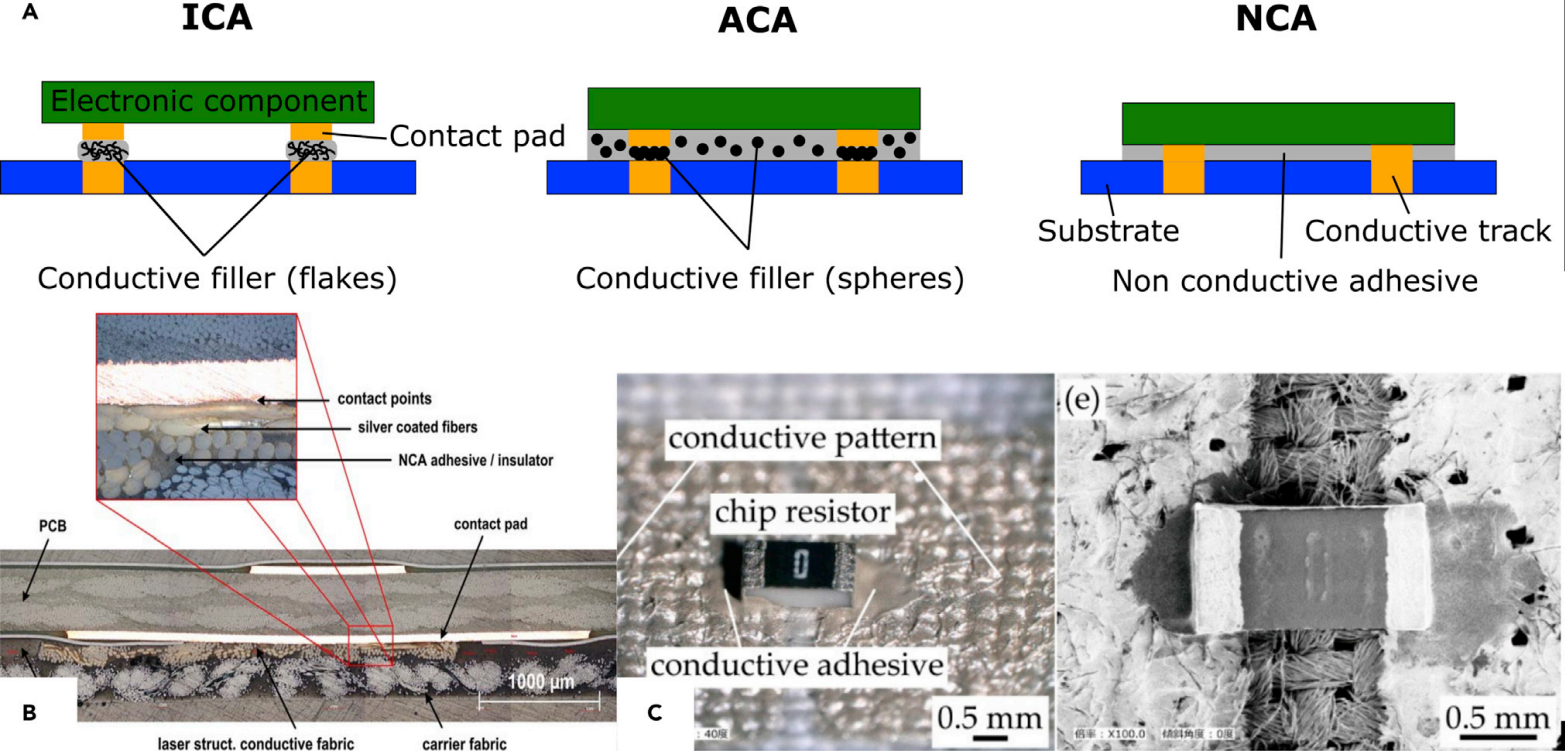
Different types of adhesives to bond electronic components on textile conductive track (A) Schematic illustrations of ACA, ICA, and NCA in flip chip bonding, (B) Bonding of a chip resistor with conductive adhesive on printed conducted tracks. Reproduced with permission from (Koshi et al., 2020b). Creative Commons CC-BY license. (C) Cross-section of a contact on a fabric circuit with embroidered conductive yarn. Reproduced with permission from (Linz et al., 2012b). Creative Commons CC-BY license.

Adhesive bonding can be considered to integrate directly the whole circuit onto textile. In 2012 Vervust et al. integrated a completely encapsulated stretchable electronic module with a thin layer of PDMS that was screen printed on textile (Vervust et al., 2012). This may lead to a local rigidity, which may result in discomfort to the wearer, but a strong mechanical robustness can be obtained. Besides, a good electrical connection can also be achieved with conductive adhesive bonding which makes it is a widely used technique for integrating electronics into textile. The different types of adhesives are schematized in Figure 11A.

a) Conductive adhesive bonding

Electrically conductive adhesives (ECA) are composed of metal particles and a polymer (epoxy) adhesive, while the metal usually being silver (Morris et al., 2005). The electrical resistivity of such adhesives is below 1 Ω cm (Mecnika et al., 2015). The physical and mechanical properties are defined by the polymeric matrix and the electrical properties are provided by the conductive fillers. Depending on their conductive filler loading level, adhesives are divided into anisotropically conductive adhesives (ACA, with a typical 3-5 mm sized conductive fillers) and isotropically conductive adhesives (ICA, with 1-10 mm sized fillers). ACA has revealed interesting electrical properties that are different in each direction depending on the pressure and temperature applied during the binding process. Hence, the conductive paths can be specifically designed without connecting other components electrically. Such an adhesive was used in a flip-chip process to mount a chip to a Cu lead frame on a fabric (Choi and Oh, 2015).

On a circuit scale, ICA was used to interconnect an RFID chip on an embroidered antenna (Chen et al., 2019). On a component scale, ICA was reported to bond an SMD chip resistor (Figure 11B) to screen-printed tracks (Koshi et al., 2020b) or a LED to nickel-plated copper wire (Tao et al., 2017). Also, it is interesting to report that conductive adhesives have been used to interconnect mechanically and electrically a two textile layer FCB to obtain a multilayered one (Lee et al., 2010).

b) Nonconductive adhesive (NCA) bonding

Whereas ACA and ICA are adhesives that are charged with conductive particles to achieve the electrical contact, NCA ensures the contact of two conductive materials by applying a mechanical force to maintain their contact. A micrograph of the resulting contact is presented in Figure 11C. Nonconductive thermoplastic polyurethane adhesive was used to bond an FR4-based electronic test module to a laser-structured fabric circuit and an embroidered one. It has reported average contact resistances of 16.5 and 28.3 mΩ, respectively (Linz et al., 2012b). It should be noted that this method enables contacting isolated conductors without having to remove the isolation in a separate step.

As described by (Holm, 1967), the contact resistance R_c_ decreases with an increasing contact force F_N_:

(Equation 18) $$Rc∼{\left(\frac{1}{F_{N}}\right)}^{n}$$

The selection of the adhesive has been extensively discussed regarding theoretical considerations of thermal expansion, relaxation behavior, and adhesion of the material. Thorough reliability tests such as temperature cycling test (JEDEC JESD22-A104-C), wash cycling test (ISO 6330) and humidity tests (JEDEC JESD22-A101-B) have been carried out (Linz et al., 2005a, 2005b, 2012b) (von Krshiwoblozki et al., 2012). Results showed that NCA-bonding is a reliable technology, under textile typical stress, to integrate electronics into textiles. Choi et al. compared the average contact resistances of the flip-chip joints processed with ACA and NCA on a heat-resistant textile fabric and obtained similar results: 5.3-10.2 mΩ and 5.5-10.1 mΩ, respectively (Choi and Oh, 2015). They also investigated specifically the durability of the NCA flip-chip and concluded that quite good reliability in the high temperature/humidity test at 85°C/85% RH can be achieved with Cu/Sn bond (Choi and Oh, 2014). Hirman et al. also tested the reliability of joints between SMD chip resistors glued by UV-curable NCA on conductive silver-coated copper threads. Those joints were found reliable for 30 washing and drying cycles (Hirman et al., 2020a).

##### Soldering

**Figure 12:**
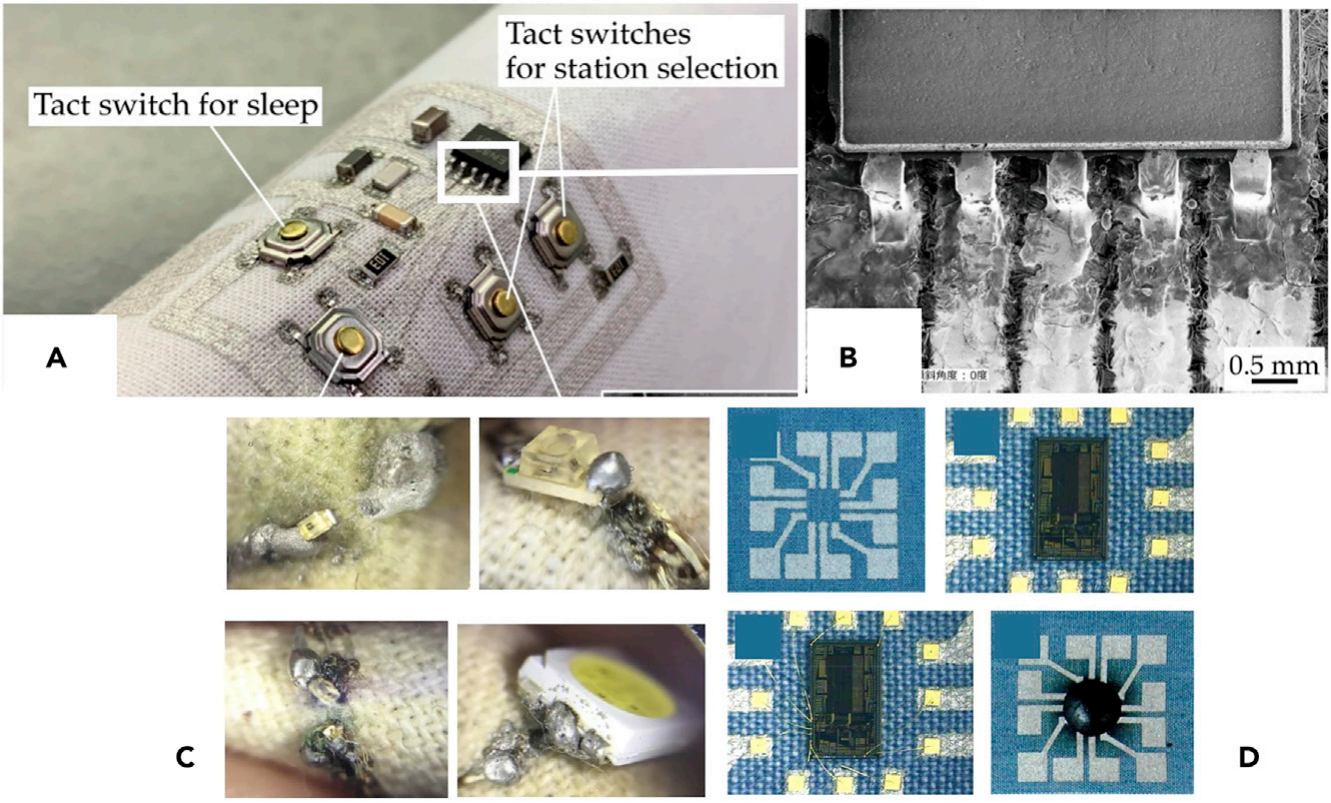
Soldering components on textile conductive tracks (A) Soldering of components on printed conductive tracks on a textile substrate. Reproduced with permission from (Koshi et al., 2020b). Creative Commons CC-BY license. (B) Micrograph of soldered component pads on screen printed conductive tracks. Reproduced with permission from (Koshi et al., 2020b). Creative Commons CC-BY license. (C) Different types of soldered interconnection failures. Reproduced with permission from (Molla et al., 2017). Copyright 2017, ACM. (D) Wire bonding on textile. Reproduced with permission from (Lee et al., 2010). Copyright 2010, IEEE.

Soldering, which consists of melting a filler metal in between two metal leads, is a standard method to obtain reliable physical and electrical connections in conventional electronics. For electronic textiles (e-textiles) the metal leads of the integrated circuit are to be connected directly with textile conductive tracks (presented in section introduction). Buechley was among the first to present solder joints on an FCB (Buechley, 2006; Buechley and Eisenberg, 2009). Nonetheless, soldering on fabric is more delicate as textiles can be damaged by the heat needed to melt the metal. Hirman et al. reported poor results with hand soldering by a soldering tip (Hirman et al., 2020b) and therefore used a tin-bismuth solder paste that melts at 155°C. Low-melt solder pastes (at 143°C (Molla et al., 2017) or at 138°C (Kallmayer and Simon, 2012)) have mostly been used to prevent any damage to the textile substrate. To enhance the durability of the soldered interconnection, Koshi et al. proposed deep permeation of patterned stretchable silver ink (Koshi et al., 2020b). The mechanical resistance of the interconnection has been carefully evaluated during cycling tensile and shear tests. Other components such as capacitors, inductors, tact switches, and a microprocessor, with a pin pitch at 1 mm, were soldered to a textile fabric in order to illustrate the suitability of this method for complete circuit fabrication. The resulting circuit is shown in Figure 12A.

Similarly, another study concluded that soldering ensures a good and durable connection of components on silver-plated copper wires (Hirman et al., 2020b). Indeed, the electrical resistance was still acceptable (<100 mΩ) after an accelerated aging of 1000 h at 85°C and 85% humidity, which is supposed to be much tougher than real-life conditions. Following a harsh tumble test, failures of soldered interconnections were reported for 215 out of 800 joints (Molla et al., 2017). Most of them were between the solder joint and the LED pad or the thread trace. Only 5 out of 215 were failures within the solder joints. The different types of solder failures are shown in Figure 12B.

In order to prevent the breakage of solder joints and preserve the conductivity of textile electrical tracks, protective materials have been considered (Molla et al., 2018). Some of them turned out to be really effective as a best-case of 0% failure rate and 0.38 Ω/m maximum increase in trace resistance were reported for component solder joints after 1000 min of washing.

To reduce the brittleness of the interconnections, ultrasonic soldering, which requires flux-free solder, has been investigated (Micus et al., 2021). Indeed, the absence of flux limits the wicking of the solder. As a consequence, the penetration of the solder into the textile substrate is reduced, resulting in more flexible joints. Soldering parameters such as the nature and quantity of the solder alloy, the temperature, time, and power of soldering are discussed.

For hard-to-reach areas, ytterbium-doped fiber laser (1064 nm) soldering has been suggested (Micus et al., 2020). This contactless method prevents any damage to the surrounding textile substrate because of a short processing time and low mechanical stress. Furthermore, it enables the use of isolated conductive yarns for the circuit as the protective tape on wires can be stripped with the laser.

It should be noted that wire bonding has also been proposed when the pitch mismatch between the integrated circuit pads and the yarns requires an interposer between the chip and the fabric (Marculescu et al., 2003). The process shown in Figure 12C has been documented by Lee et al. (2010).

##### Comparison between methods

Overviews of existing jointing technologies in the electronics and textile worlds can be found in other articles (Locher and Sefar, 2013) and (Mecnika et al., 2015). In terms of electrical resistance, studies have reported that electrical resistance is lower for soldered chip resistors (Micus et al., 2020), (Hirman et al., 2020a) than glued ones. However, by comparing the reported values for glue (Table 6) and solder (Table 7) one may see that both contact resistance are in the same order of magnitude. In terms of electrical properties, both solder and glue are promising candidates whose application may depend on the substrate temperature tolerance.

**Table 6:** Synthesis of methods reported for gluing electronic components on textile conductive tracks

Adhesive type	Conductive tracks	Components	Curing process	Contact resistance (mΩ)	References
ICA	Screen-printed stretchable silver ink	Chip resistors (0 Ω)	Room temperature for 1 h	610–1140	(Koshi et al., 2020b)
ICA conductive silver	Nickel-plated copper wire	LED	–	–	(Tao et al., 2017)
ACA	Cu Lead	Si chip	Flip-chip bonding by holding at 160°C and 100 MPa for 1 min	5.3–10.2	(Choi and Oh, 2015)
NCA				5.5–10.1	
NCA				5.4–10.8	(Choi and Oh, 2014)
NCA TPU film	Conductive fabric and embroidered conductive yarn (fibers metallized with nanosilver)	Test module	195°C for 30 s Pressure: 0.7 N/mm^2^	Resistance for path + joints: • 23 Ω for fabric circuits) • 9 Ω for embroidered circuits.	(Linz et al., 2012b)
NCA	Silver-plated copper sewing thread	Chip resistors (0 Ω)	UV light + mechanical pressure	30	(Hirman et al., 2020a)
NCA	Litz wires with Aramid-Core (LWAC) silver coated yarn	Test vehicle: Micro PCB with 4 LED and IC driving	200°C Pressure: 1.9–3.1 N/mm^2^ depending on the fabric structure	• <2 for insulated and the noninsulated LWAC • 1–25 for silver yarn	(von Krshiwoblozki et al., 2012)

**Table 7:** Methods reported for soldering electronic components on textile conductive tracks

Solder paste	Conductive track	Components	Soldering process	Contact resistance (mΩ)	References
Sn_0.63_Pb_0.37_ (1 mg)	Screen printed stretchable silver ink	Chip resistors (0 Ω)	Iron @ 250–300°C	490–1050	(Koshi et al., 2020b)
ChipQuick	Embroidered silver conductive thread	LED	Hot air @ 143°C	–	(Molla et al., 2017)
Sn_0.63_Pb_0.37_ Sn_0.42_Bi_0.58_	Silver-plated copper sewing thread	Chip resistors (0 Ω)	Hot air @ 155°C	20	(Hirman et al., 2020b)
Loctite GC10 Sn_0.965_Ag_0.03_Cu_0.005_	Nickel-plated copper wire	LED	Hot air @ 230°C	–	(Tao et al., 2017)
Tin-bismuth solder paste	Silver-plated polyamide	Interposer	Hot air @ 138°C	–	(Kallmayer and Simon, 2012)
Sn_0.965_Ag_0.03_Cu_0.005_ Sn_0.955_Ag_0.038_Cu_0.007_ Sn_0.42_Bi_0.576_Ag_0.004_ Sn_0.4347_Bi_0.5585_Ag_0.0068_	Lacquer-insulated copper strands	Small PCB	Laser	10	(Micus et al., 2020)
(Flux-free solder pastes) Sn_0.955_Ag_0.038_Cu_0.007_ Sn_0.997_Cu_0.003_ Sn_0.97_Ag_0.03_	Silver-coated copper strand	Small PCB	Ultrasound @350–390°C	6–8	(Micus et al., 2021)

When working on a heat-sensitive substrate, such as textile, glues are more promising as some of them may be applied and cured at room temperature, thus preventing any damage to the textile. In a more general sense, glues may be processed with a temperature ≤200°C where solders mostly require a temperature over 200°C. One exception is SnBi alloys that exhibit soldering temperature of 138°C. It should also be kept in mind that not every metal can be welded using tin alloys where glues may be effective on most solid surfaces.

The mechanical strength of joints with conductive adhesives is relatively low owing to the high mass fraction of the conductive particles in the adhesive system. These particles generally weaken the adhesive (Sancaktar and Bai, 2011). Cycling tensile tests on glued or soldered components on textile show that changes in resistance were 3.0-4.2 times lower for the samples soldered (Koshi et al., 2020b). In this particular experiment, components were connected to screen printing silver lines on textile. It should be noted that the deep permeation of the ink into the textile ensures greater durability of the electrical contact (Koshi et al., 2020b). Only Tao et al. reported better washability for glued components than soldered (Tao et al., 2017). In this study, SMD LED were interconnected to a nickel-platted copper wire. In this peculiar case, solder joint is brittle for nickel which resulted in a solder interconnection less reliable than the bonded one.

## Conclusions

This work aimed to bring a global comprehension of wearable textile tribogenerators for embedded applications through different aspects: a comprehensive study of triboelectricity phenomenon, the textile integration of needed materials, and the electronic integration of power transfer circuits onto textiles.

A wide variety of T-TENG have been explored at the step of the proof of concept, but several key points have to be reunited in the design of one device in order to maximize the chances to be widely adopted in a short span of time. The T-TENG should be fabricated with processes that are compatible with or already integrated into the textile value chain and be composed of materials that are already available or easily embeddable in the textile value chain. The resistance to long-term mechanical deformations and resistance to domestic washing is crucial, especially if the T-TENG is integrated to pieces of clothes that are close to the body and need to be frequently washed. The drastic performance loss of most TENGs in humid environment can be problematic in the case of integration for sportswear applications, such as the risk of rain in the case of outer clothing and influence of sweat if integrated next to the body (most of the friction area in the body are also areas where sweat appears easily). This parameter is to be taken into account early in the characterization tests in order to prevent dysfunctions of the T-TENG at higher technology readiness levels when going from lab tests to real condition tests. The size and imperceptibility of the T-TENG are essential, optimization of the structure and the global design of integration in the garment will be of utter importance once it will be ready to be on the market. Finally, a standard for the measurement of performances is needed in order to be able to compare the efficiency of different T-TENG systems (same intensity of trigger for example).

Maxwell’s approach seems to be efficient for TENGs modeling. However, some numerical issues appear for human motions at low frequency (<3 Hz). In the future, it will be suitable to solve this problem with a new approach. The imperceptible integration of textiles of the energy transfer circuit for the conversion and storage of the charges generated by the T-TENG requires the reliable interconnection of various electronic components (passive components, microcontroller, battery, and so forth) on conductive tracks. Conductive tracks with low resistivity and lateral resolution compatible with the fine pitch of a microcontroller can be obtained by printing and laser ablation techniques. Nowadays, screen printing is probably the most successful and mature technique. Bonding and soldering are the two major processes used to achieve a high level of interconnection reliability. Robustness to mechanical stress and washability remain challenges and require the use of stretchable inks and an encapsulating protective layer as well.

## References

[bib1] Ahmed A., Hassan I., El-Kady M.F., Radhi A., Jeong C.K., Selvaganapathy P.R., Zu J., Ren S., Wang Q., Kaner R.B. (2019). Integrated triboelectric nanogenerators in the era of the internet of things. Adv. Sci..

[bib2] Alicki R., Jenkins A. (2020). Quantum theory of triboelectricity. Phys. Rev. Lett..

[bib5] Briedis U., Valisevskis A., Grecka M. (2017). Development of a smart garment prototype with enuresis alarm using an embroidery-machine-based technique for the integration of electronic components. Procedia Comput. Sci..

[bib6] Buechley L. (2006). 2006 10th IEEE International Symposium on Wearable Computers. Presented at the 2006 10th IEEE International Symposium on Wearable Computers.

[bib7] Buechley L., Eisenberg M. (2009). Fabric PCBs, electronic sequins, and socket buttons: techniques for e-textile craft. Pers. Ubiquitous Comput..

[bib8] Cai Z., Luo K., Liu C., Li J. (2017). Design of a smart ECG garment based on conductive textile electrode and flexible printed circuit board. Technol. Health Care.

[bib9] Catarino A., Rocha A.M., Abreu M.J., Derogarian F., Da Silva J., Ferreira J., Tavares V., Correia M., Dias R. (2013). E-legging for monitoring the human locomotion patterns. J. Text. Eng..

[bib10] Chen J., Guo H., Pu X., Wang X., Xi Y., Hu C. (2018). Traditional weaving craft for one-piece self-charging power textile for wearable electronics. Nano Energy.

[bib11] Chen X., Ukkonen L., Virkki J. (2019). Reliability evaluation of wearable radio frequency identification tags: design and fabrication of a two-part textile antenna. Text. Res. J..

[bib12] Chen J., Wang J., Xuan W., Dong S., Luo J. (2020). Universal triboelectric nanogenerator simulation based on dynamic finite element method model. Sensors.

[bib13] Chen C., Guo H., Chen L., Wang Y.-C., Pu X., Yu W., Wang F., Du Z., Wang Z.L. (2020). Direct current fabric triboelectric nanogenerator for biomotion energy harvesting. ACS Nano.

[bib14] Chen A., Zhang C., Zhu G., Wang Z.L. (2020). Polymer materials for high-performance triboelectric nanogenerators. Adv. Sci..

[bib15] Choi D., Kim D.W., Yoo D., Cha K.J., La M., Kim D.S. (2017). Spontaneous occurrence of liquid-solid contact electrification in nature: toward a robust triboelectric nanogenerator inspired by the natural lotus leaf. Nano Energy.

[bib16] Choi J.-Y., Oh T.S. (2015). Contact resistance comparison of flip-chip joints produced with anisotropic conductive adhesive and nonconductive adhesive for smart textile applications. Mater. Trans..

[bib17] Choi J.-Y., Oh T.S. (2014). Contact resistance of flip-chip joints in wearable electronic textiles. J. Electron. Mater..

[bib18] Cong Z., Guo W., Guo Z., Chen Y., Liu M., Hou T., Pu X., Hu W., Wang Z.L. (2020). Stretchable coplanar self-charging power textile with resist-dyeing triboelectric nanogenerators and microsupercapacitors. ACS Nano.

[bib19] Coyle S., Morris D., Lau K.-T., Diamond D., Moyna N. (2009). 2009 Sixth International Workshop on Wearable and Implantable Body Sensor Networks. Presented at the Implantable Body Sensor Networks Conference (BSN).

[bib20] Cui N., Liu J., Gu L., Bai S., Chen X., Qin Y. (2015). Wearable triboelectric generator for powering the portable electronic devices. ACS Appl. Mater. Interfaces.

[bib22] Dils C., Werft L., Walter H., Zwanzig M., von Krshiwoblozki M., Schneider-Ramelow M. (2019). Investigation of the mechanical and electrical properties of elastic textile/polymer composites for stretchable electronics at quasi-static or cyclic mechanical loads. Materials.

[bib23] Dong K., Deng J., Ding W., Wang A.C., Wang P., Cheng C., Wang Y.-C., Jin L., Gu B., Sun B., Wang Z.L. (2018). Versatile core-sheath yarn for sustainable biomechanical energy harvesting and real-time human-interactive sensing. Adv. Energy Mater..

[bib24] Dong K., Deng J., Zi Y., Wang Y.-C., Xu C., Zou H., Ding W., Dai Y., Gu B., Sun B., Wang Z.L. (2017). 3D orthogonal woven triboelectric nanogenerator for effective biomechanical energy harvesting and as self-powered active motion sensors. Adv. Mater..

[bib25] Dong K., Hu Y., Yang J., Kim S.-W., Hu W., Wang Z.L. (2021). Smart textile triboelectric nanogenerators: current status and perspectives. MRS Bull..

[bib26] Dong K., Wang Y.-C., Deng J., Dai Y., Zhang S.L., Zou H., Gu B., Sun B., Wang Z.L. (2017). A highly stretchable and washable all-yarn-based self-charging knitting power textile composed of fiber triboelectric nanogenerators and supercapacitors. ACS Nano.

[bib27] Dong S., Xu F., Sheng Y., Guo Z., Pu X., Liu Y. (2020). Seamlessly knitted stretchable comfortable textile triboelectric nanogenerators for E-textile power sources. Nano Energy.

[bib28] Electromagnetic Field Shielding Fabrics [WWW Document], 2021. URL https://www.lessemf.com/fabric.html#207 (accessed 11.16.21).

[bib29] Escobedo P., de Pablos-Florido J., Carvajal M.A., Martínez-Olmos A., Capitán-Vallvey L.F., Palma A.J. (2020). The effect of bending on laser-cut electro-textile inductors and capacitors attached on denim as wearable structures. Text. Res. J..

[bib30] Fan F.-R., Tian Z.-Q., Lin Wang Z. (2012). Flexible triboelectric generator. Nano Energy.

[bib31] Feshanjerdi M., Malekan A. (2019). Contact electrification between randomly rough surfaces with identical materials. J. Appl. Phys..

[bib32] Garnier B., Mariage P., Rault F., Cochrane C., Koncar V. (2021). Electronic-components less fully textile multiple resonant combiners for body-centric near field communication. Sci. Rep..

[bib33] Gaubert V., Gidik H., Koncar V. (2020). Boxer underwear incorporating textile moisture sensor to prevent nocturnal enuresis. Sensors.

[bib34] Ghaffarinejad A., Hasani J.Y., Hinchet R., Lu Y., Zhang H., Karami A., Galayko D., Kim S.-W., Basset P. (2018). A conditioning circuit with exponential enhancement of output energy for triboelectric nanogenerator. Nano Energy.

[bib35] Gong W., Hou C., Zhou J., Guo Y., Zhang W., Li Y., Zhang Q., Wang H. (2019). Continuous and scalable manufacture of amphibious energy yarns and textiles. Nat. Commun..

[bib36] Grimmelsmann N., Martens Y., Schäl P., Meissner H., Ehrmann A. (2016). Mechanical and electrical contacting of electronic components on textiles by 3D printing. Proc. Technol..

[bib37] Guan X., Xu B., Huang J., Jing T., Gao Y. (2022). Fiber-shaped stretchable triboelectric nanogenerator with a novel synergistic structure of opposite Poisson’s ratios. Chem. Eng. J..

[bib38] Henniker J. (1962). Triboelectricity in polymers. Nature.

[bib39] Hirman M., Navratil J., Steiner F., Hamacek A. (2020). 2020 IEEE 8th Electronics System-Integration Technology Conference (ESTC). Presented at the 2020 IEEE 8th Electronics System-Integration Technology Conference (ESTC).

[bib40] Hirman M., Navratil J., Steiner F., Hamacek A. (2020). 2020 43rd International Spring Seminar on Electronics Technology (ISSE). Presented at the 2020 43rd International Spring Seminar on Electronics Technology (ISSE).

[bib41] Holm R. (1967).

[bib42] Hong H., Hu J., Yan X. (2019). UV curable conductive ink for the fabrication of textile-based conductive circuits and wearable UHF RFID tags. ACS Appl. Mater. Interfaces.

[bib43] Huang T., Zhang J., Yu B., Yu H., Long H., Wang H., Zhang Q., Zhu M. (2019). Fabric texture design for boosting the performance of a knitted washable textile triboelectric nanogenerator as wearable power. Nano Energy.

[bib44] Jin H., Matsuhisa N., Lee S., Abbas M., Yokota T., Someya T. (2017). Enhancing the performance of stretchable conductors for E-textiles by controlled ink permeation. Adv. Mater..

[bib45] Kallmayer C., Simon E. (2012). International Multi-Conference on Systems, Signals & Devices. Presented at the 2012 IEEE 9th International Multi-Conference on Systems, Signals and Devices (SSD).

[bib46] Kaponig M., Mölleken A., Nienhaus H., Möller R. (2021). Dynamics of contact electrification. Sci. Adv..

[bib47] Karaguzel B., Merritt C.R., Kang T., Wilson J.M., Nagle H.T., Grant E., Pourdeyhimi B. (2009). Flexible, durable printed electrical circuits. J. Textil. Inst..

[bib48] Karim N., Afroj S., Malandraki A., Butterworth S., Beach C., Rigout M., Novoselov K.S., Casson A.J., Yeates S.G. (2017). All inkjet-printed graphene-based conductive patterns for wearable e-textile applications. J. Mater. Chem. C.

[bib49] Karim N., Afroj S., Tan S., Novoselov K.S., Yeates S.G. (2019). All inkjet-printed graphene-silver composite ink on textiles for highly conductive wearable electronics applications. Sci. Rep..

[bib50] Kim H., Kim Y., Kim B., Yoo H.-J. (2009). 2009 Sixth International Workshop on Wearable and Implantable Body Sensor Networks. Presented at the Implantable Body Sensor Networks Conference (BSN).

[bib52] Kim I., Shahariar H., Ingram W.F., Zhou Y., Jur J.S. (2019). Inkjet process for conductive patterning on textiles: maintaining inherent stretchability and breathability in knit structures. Adv. Funct. Mater..

[bib53] Kiourti A., Volakis J.L. (2015). High-geometrical-accuracy embroidery process for textile antennas with fine details. IEEE Antennas Wirel. Propag. Lett..

[bib54] Koshi T., Nomura K.I., Yoshida M. (2019). Requirements for durability improvement of conductive patterns permeated in textiles under cyclic tensile deformation. Micromachines.

[bib55] Koshi T., Nomura K.I., Yoshida M. (2020). Electrical characterization of a double-layered conductive pattern with different crack configurations for durable E-textiles. Micromachines.

[bib56] Koshi T., Nomura K.I., Yoshida M. (2020). Electronic component mounting for durable E-textiles: direct soldering of components onto textile-based deeply permeated conductive patterns. Micromachines.

[bib57] Krykpayev B., Farooqui M.F., Bilal R.M., Vaseem M., Shamim A. (2017). A wearable tracking device inkjet-printed on textile. Microelectronics J..

[bib58] Kulbago B.J., Chen J. (2020). Nonlinear potential field in contact electrification. J. Electrostat..

[bib59] Kwak S.S., Kim H., Seung W., Kim J., Hinchet R., Kim S.-W. (2017).

[bib60] Lacks D.J. (2012). The unpredictability of electrostatic charging. Angew. Chem. Int. Ed. Engl..

[bib61] Lacks D.J., Shinbrot T. (2019). Long-standing and unresolved issues in triboelectric charging. Nat. Rev. Chem.

[bib62] Li Q., Ran Z., Ding X., Wang X. (2019). Fabric circuit board connecting to flexible sensors or rigid components for wearable applications. Sensors.

[bib63] Li S., Nie J., Shi Y., Tao X., Wang F., Tian J., Lin S., Chen X., Wang Z.L. (2020). Contributions of different functional groups to contact electrification of polymers. Adv. Mater..

[bib65] Lin S., Xu L., Xu C., Chen X., Wang A.C., Zhang B., Lin P., Yang Y., Zhao H., Wang Z.L. (2019). Electron transfer in nanoscale contact electrification: effect of temperature in the metal–dielectric case. Adv. Mater..

[bib66] Linz T., Kallmayer C., Aschenbrenner R., Reichl H. (2005). Ninth IEEE International Symposium on Wearable Computers (ISWC’05). Presented at the Proceedings. Ninth IEEE International Symposium on Wearable Computers.

[bib67] Linz T., Kallmayer C., Aschenbrenner R., Reichl H. (2005). http://publica.fraunhofer.de/eprints/urn_nbn_de_0011-n-1205423.pdf.

[bib68] Linz T., Vieroth R., Dils C., Koch M., Braun T., Becker K.F., Kallmayer C., Hong S.M. (2008). Embroidered interconnections and encapsulation for electronics in textiles for wearable electronics applications. Adv. Sci. Technol..

[bib69] Linz T., Simon E., Walter H. (2010). 3rd Electronics System Integration Technology Conference ESTC. Presented at the 2010 3rd Electronic System-Integration Technology Conference (ESTC).

[bib70] Linz T., Simon E.P., Walter H. (2012). Modeling embroidered contacts for electronics in textiles. J. Textil. Inst..

[bib71] Linz T., von Krshiwoblozki M., Walter H., Foerster P. (2012). Contacting electronics to fabric circuits with nonconductive adhesive bonding. J. Textil. Inst..

[bib72] Liu J., Gu L., Cui N., Bai S., Liu S., Xu Q., Qin Y., Yang R., Zhou F. (2019). Core-shell fiber-based 2D woven triboelectric nanogenerator for effective motion energy harvesting. Nanoscale Res. Lett..

[bib73] Locher I., Sefar A.G. (2013). Multidisciplinary Know-How for Smart-Textiles Developers.

[bib74] Locher I., Troster G. (2007). Fundamental building blocks for circuits on textiles. IEEE Trans. Adv. Packag..

[bib75] Lu M., Yin W., Peyton A., Qu Z., Meng X., Xie Y., Zhao P., Luo J., Zhao Q., Tao Y. (2019). A model for the triboelectric nanogenerator with inductive load and its energy boost potential. Nano Energy.

[bib76] Ma L., Wu R., Patil A., Yi J., Liu D., Fan X., Sheng F., Zhang Y., Liu S., Shen S. (2021). Acid and alkali-resistant textile triboelectric nanogenerator as a smart protective suit for liquid energy harvesting and self-powered monitoring in high-risk environments. Adv. Funct. Mater..

[bib77] Mao Y., Li Y., Xie J., Liu H., Guo C., Hu W. (2021). Triboelectric nanogenerator/supercapacitor in-one self-powered textile based on PTFE yarn wrapped PDMS/MnO2NW hybrid elastomer. Nano Energy.

[bib78] Marculescu D., Marculescu R., Zamora N.H., Stanley-Marbell P., Khosla P.K., Park S., Jayaraman S., Jung S., Lauterbach C., Weber W. (2003). Electronic textiles: a platform for pervasive computing. Proc. IEEE.

[bib80] Mecnika V., Scheulen K., Anderson C.F., Hörr M., Breckenfelder C. (2015). Electronic Textiles.

[bib81] Mehmood A., Qureshi S., He H., Chen X., Ahmed S., Merilampi S., Raumonen P., Ukkonen L., Virkki J. (2019). 2019 IEEE 7th International Conference on Serious Games and Applications for Health (SeGAH). Presented at the 2019 IEEE 7th International Conference on Serious Games and Applications for Health (SeGAH).

[bib82] Meng J., Guo Z.H., Pan C., Wang L., Chang C., Li L., Pu X., Wang Z.L. (2021). Flexible textile direct-current generator based on the tribovoltaic effect at dynamic metal-semiconducting polymer interfaces. ACS Energy Lett..

[bib83] Micus S., Haupt M., Gresser G.T. (2021). Automatic joining of electrical components to smart textiles by ultrasonic soldering. Sensors.

[bib84] Micus S., Haupt M., Gresser G.T. (2020). Soldering electronics to smart textiles by pulsed Nd:YAG laser. Materials.

[bib85] Molla M.T.I., Compton C., Dunne L.E. (2018). Proceedings of the 2018 ACM International Symposium on Wearable Computers. Presented at the UbiComp ’18: The 2018 ACM International Joint Conference on Pervasive and Ubiquitous Computing.

[bib86] Molla M.T.I., Goodman S., Schleif N., Berglund M.E., Zacharias C., Compton C., Dunne L.E. (2017). Proceedings of the 2017 ACM International Symposium on Wearable Computers. Presented at the UbiComp ’17: The 2017 ACM International Joint Conference on Pervasive and Ubiquitous Computing.

[bib87] Morris J.E., Lee J., Liu J. (2005). Polytronic 2005 - 5th International Conference on Polymers and Adhesives in Microelectronics and Photonics. Presented at the Polytronic 2005 - 5th International Conference on Polymers and Adhesives in Microelectronics and Photonics.

[bib88] Mule A.R., Dudem B., Patnam H., Graham S.A., Yu J.S. (2019). Wearable single-electrode-mode triboelectric nanogenerator via conductive polymer-coated textiles for self-power electronics. ACS Sustain. Chem. Eng..

[bib89] Nechyporchuk O., Yu J., Nierstrasz V.A., Bordes R. (2017). Cellulose Nanofibril-Based Coatings of Woven Cotton Fabrics for Improved Inkjet Printing with a Potential in E-Textile Manufacturing 22.

[bib90] Niu S., Wang S., Lin L., Liu Y., Zhou Y.S., Hu Y., Wang Z.L. (2013). Theoretical study of contact-mode triboelectric nanogenerators as an effective power source. Energy Environ. Sci..

[bib91] Ojstršek A., Plohl O., Gorgieva S., Kurečič M., Jančič U., Hribernik S., Fakin D. (2021). Metallisation of textiles and protection of conductive layers: an overview of application techniques. Sensors.

[bib92] Paiva A., Catarino A., Carvalho H., Postolache O., Postolache G., Ferreira F., Machado J., Soares F., Veiga G. (2019). Innovation, Engineering and Entrepreneurship, Lecture Notes in Electrical Engineering.

[bib93] Pan S., Zhang Z. (2017). Triboelectric effect: a new perspective on electron transfer process. J. Appl. Phys..

[bib95] Paradiso R., Caldani L., Pacelli M. (2014). Wearable Sensors.

[bib96] Pawlak R., Korzeniewska E., Koneczny C., Hałgas B. (2017). Properties of thin metal layers deposited on textile composites by using the Pvd method for textronic applications. Autex Res. J..

[bib97] Pawlak R., Lebioda M., Tomczyk M., Rymaszewski J., Korzeniewska E., Walczak M. (2018). Modelling and applications of conductive elements on textile materials. COMPEL - Int. J. Comput. Math. Electr. Electron. Eng..

[bib99] Poupyrev I., Gong N.-W., Fukuhara S., Karagozler M.E., Schwesig C., Robinson K.E. (2016). Proceedings of the 2016 CHI Conference on Human Factors in Computing Systems - CHI ’16. Presented at the the 2016 CHI Conference.

[bib100] Pu X., Li L., Song H., Du C., Zhao Z., Jiang C., Cao G., Hu W., Wang Z.L. (2015). A self-charging power unit by integration of a textile triboelectric nanogenerator and a flexible lithium-ion battery for wearable electronics. Adv. Mater..

[bib101] Pu X., Liu M., Li L., Zhang C., Pang Y., Jiang C., Shao L., Hu W., Wang Z.L. (2016). Efficient charging of Li-ion batteries with pulsed output current of triboelectric nanogenerators. Adv. Sci..

[bib102] Righetti X., Thalmann D. (2010). Melecon 2010 - 2010 15th IEEE Mediterranean Electrotechnical Conference. Presented at the Melecon 2010 - 2010 15th IEEE Mediterranean Electrotechnical Conference.

[bib103] Rotzler S., Kallmayer C., Dils C., von Krshiwoblozki M., Bauer U., Schneider-Ramelow M. (2020). Improving the washability of smart textiles: influence of different washing conditions on textile integrated conductor tracks. J. Textil. Inst..

[bib104] Sancaktar E., Bai L. (2011). Electrically conductive epoxy adhesives. Polymers.

[bib105] Scheulen K., Schwarz A., Jockenhoevel S. (2013). Proceedings of the 17th Annual International Symposium on International Symposium on Wearable Computers - ISWC ’13. Presented at the the 17th annual international symposium.

[bib106] Schwarz A., Van Langenhove L., Guermonprez P., Deguillemont D. (2010). A roadmap on smart textiles. Textil. Prog..

[bib107] Seager R.D., Chauraya A., Zhang S., Whittow W., Vardaxoglou Y. (2013). Flexible radio frequency connectors for textile electronics. Electron. Lett..

[bib108] Lee S., Kim B., Roh T., Hong S., Yoo H.-J. (2010). International Symposium on Wearable Computers (ISWC) 2010. Presented at the 2010 International Symposium on Wearable Computers (ISWC).

[bib109] Seung W., Gupta M.K., Lee K.Y., Shin K.-S., Lee J.-H., Kim T.Y., Kim S., Lin J., Kim J.H., Kim S.-W. (2015). Nanopatterned textile-based wearable triboelectric nanogenerator. ACS Nano.

[bib110] Shahariar H., Kim I., Soewardiman H., Jur J.S. (2019). Inkjet printing of reactive silver ink on textiles. ACS Appl. Mater. Interfaces.

[bib111] Shao J., Jiang T., Wang Z. (2020). Theoretical foundations of triboelectric nanogenerators (TENGs). Sci. China Technol. Sci..

[bib112] Shao J., Liu D., Willatzen M., Wang Z.L. (2020). Three-dimensional modeling of alternating current triboelectric nanogenerator in the linear sliding mode. Appl. Phys. Rev..

[bib113] Shao J., Willatzen M., Wang Z.L. (2020). Theoretical modeling of triboelectric nanogenerators (TENGs). J. Appl. Phys..

[bib114] Shao J., Willatzen M., Shi Y., Wang Z.L. (2019). 3D mathematical model of contact-separation and single-electrode mode triboelectric nanogenerators. Nano Energy.

[bib115] Shen X., Wang A.E., Sankaran R.M., Lacks D.J. (2016). First-principles calculation of contact electrification and validation by experiment. J. Electrostat..

[bib116] Simon E.P., Kallmayer C., Aschenbrenner R., Lang K.-D. (2011). Proceedings of the 18th European Microelectronics & Packaging Conference.

[bib117] Stempien Z., Pawlak R., Korzeniewska E. (2016). 2016 13th Selected Issues of Electrical Engineering and Electronics (WZEE). Presented at the 2016 13th Selected Issues of Electrical Engineering and Electronics (WZEE).

[bib118] Stempien Z., Rybicki E., Patykowska A., Rybicki T., Szynkowska M. (2018). Shape-programmed inkjet-printed silver electro-conductive layers on textile surfaces. J. Ind. Text..

[bib119] Stoppa M., Chiolerio A. (2014). Wearable electronics and smart textiles: a critical review. Sensors.

[bib120] Sugathan M., Hendry M.D. (2017). Market forecasts and personal adoption of smart textiles in fitness sector. Int. J. Technol. Diffus..

[bib121] Suo G., Wang X., Li J., Yu Y., Zhang Z., Wang S., Zhao P. (2016). Piezoelectric and triboelectric dual effects in mechanical energy harvesting using BaTiO3/polydimethylsiloxane composite film. ACS Appl. Mater.Interfaces.

[bib122] Šutka A., Sherrell P.C., Shepelin N.A., Lapčinskis L., Mālnieks K., Amanda V.E. (2020). Measuring piezoelectric output—fact or friction?. Adv. Mater..

[bib123] Tao X., Koncar V., Huang T.-H., Shen C.-L., Ko Y.-C., Jou G.-T. (2017). How to make reliable, washable, and wearable textronic devices. Sensors.

[bib124] Vervust T., Buyle G., Bossuyt F., Vanfleteren J. (2012). Integration of stretchable and washable electronic modules for smart textile applications. J. Textil. Inst..

[bib125] Vick F.A. (1953). Theory of contact electrification. Br. J. Appl. Phys..

[bib126] von Krshiwoblozki M., Linz T., Neudeck A., Kallmayer C. (2012). Electronics in textiles – adhesive bonding technology for reliably embedding electronic modules into textile circuits. Adv. Sci. Technol..

[bib127] Wang A.C., Zhang B., Xu C., Zou H., Lin Z., Wang Z.L. (2020). Unraveling temperature-dependent contact electrification between sliding-mode triboelectric pairs. Adv. Funct. Mater..

[bib128] Wang Z.L., Wang A.C. (2019). On the origin of contact-electrification. Mater. Today.

[bib129] Wu M., Li J. (2021). Sliding ferroelectricity in 2D van der Waals materials: related physics and future opportunities. Proc. Natl. Acad. Sci. USA.

[bib130] Xi F., Pang Y., Li W., Jiang T., Zhang L., Guo T., Liu G., Zhang C., Wang Z.L. (2017). Universal power management strategy for triboelectric nanogenerator. Nano Energy.

[bib131] Xu C., Zi Y., Wang A.C., Zou H., Dai Y., He X., Wang P., Wang Y.-C., Feng P., Li D., Wang Z.L. (2018). On the electron-transfer mechanism in the contact-electrification effect. Adv. Mater..

[bib132] Xu F., Dong S., Liu G., Pan C., Guo Z.H., Guo W., Li L., Liu Y., Zhang C., Pu X., Wang Z.L. (2021). Scalable fabrication of stretchable and washable textile triboelectric nanogenerators as constant power sources for wearable electronics. Nano Energy.

[bib133] Yang Y., Xie L., Wen Z., Chen C., Chen X., Wei A., Cheng P., Xie X., Sun X. (2018). Coaxial triboelectric nanogenerator and supercapacitor fiber-based self-charging power fabric. ACS Appl. Mater. Interfaces.

[bib134] Yu A., Pu X., Wen R., Liu M., Zhou T., Zhang K., Zhang Y., Zhai J., Hu W., Wang Z.L. (2017). Core–shell-yarn-based triboelectric nanogenerator textiles as power cloths. ACS Nano.

[bib135] Zhang G., Quetzeri-Santiago M.A., Stone C.A., Botto L., Castrejón-Pita J.R. (2018). Droplet impact dynamics on textiles. Soft Matter.

[bib136] Zhang P., Zhang W., Zhang H. (2021). A high-performance textile-based triboelectric nanogenerator manufactured by a novel brush method for self-powered human motion pattern detector. Sustain. Energy Technol. Assessments.

[bib137] Zhong J., Zhang Y., Zhong Q., Hu Q., Hu B., Wang Z.L., Zhou J. (2014). Fiber-based generator for wearable electronics and mobile medication. ACS Nano.

[bib138] Zhou T., Zhang C., Han C.B., Fan F.R., Tang W., Wang Z.L. (2014). Woven structured triboelectric nanogenerator for wearable devices. ACS Appl. Mater. Interfaces.

[bib139] Zhou Y., Deng W., Xu J., Chen J. (2020). Engineering materials at the nanoscale for triboelectric nanogenerators. Cell Rep. Phys. Sci..

[bib141] Zou H., Zhang Y., Guo L., Wang P., He X., Dai G., Zheng H., Chen C., Wang A.C., Xu C., Wang Z.L. (2019). Quantifying the triboelectric series. Nat. Commun..

[bib142] Zou H., Guo L., Xue H., Zhang Y., Shen X., Liu X., Wang P., He X., Dai G., Jiang P. (2020). Quantifying and understanding the triboelectric series of inorganic non-metallic materials. Nat. Commun..

